# PCIPG: A comprehensive framework for protein complex identification based on a probabilistic graphical model

**DOI:** 10.1371/journal.pcbi.1014698

**Published:** 2026-09-03

**Authors:** Yixiang Huang, Lei Yang, Jiudong Wang, Xinqi Gong

**Affiliations:** 1 School of Mathematics, Renmin University of China, Beijing, China; 2 School of Life Science, Beijing Institute of Technology, Beijing, China; Tel Aviv University, ISRAEL

## Abstract

Protein complexes are molecular machines that execute essential cellular functions, but their computational identification remains challenging. Existing protein complex identification methods largely rely on PPI network topology, functional annotations, or protein-level biochemical evidence. Although these approaches have recovered many biologically meaningful assemblies, they are often sensitive to incomplete or noisy interactomes and provide limited mechanistic insight into the residue- and interface-level determinants of complex formation. In particular, conventional PPI-based graph representations indicate whether proteins are associated, but usually ignore how protein subunits physically interact through spatially organized residues and structural interfaces. These limitations motivate the development of computational frameworks that connect residue-scale structural cues with interactome-scale organization. Here we present PCIPG, a multi-scale probabilistic graph framework that jointly models residues, proteins, interactions and complexes. PCIPG encodes residue-level physicochemical descriptors on intra-chain contact maps, screens informative residues to construct structure-aware protein representations and propagates these representations over the PPI graph to infer a protein–complex membership matrix. To couple complex membership with sparse interaction evidence, PCIPG reconstructs the network using a zero-inflated Bernoulli–Exponential likelihood, providing a principled learning signal under missing-edge and noise regimes. Across five Saccharomyces cerevisiae benchmarks, PCIPG achieved higher average F1 and Acc than the representative baseline methods included in this study, with average improvements of 11.46% and 3.64%, respectively. On the evaluated human interactomes, PCIPG achieved the highest F1 score among the compared methods on HCT116 and HEK293T, whereas its performance on HuRI was below that of AdaPPI and ClusterONE. Embedding-guided interaction completion improved PCIPG’s performance relative to its results on the corresponding original human PPI networks. Beyond complex calling, PCIPG supports core–module mining by recovering known cores and delineating coherent accessory modules within assemblies; several predictions match previously reported functional entities, including TRAPPII- and PCNA-loading-factor–related complexes. At the residue level, residues prioritized by PCIPG show increased overlap with experimentally defined protein-binding interfaces in the evaluated structures. In a computational CFTR case study, the model generated state-dependent interaction predictions that partially overlapped with experimentally profiled wild-type and ΔF508 interaction networks. Together, PCIPG bridges residue-scale structural cues with interactome-scale organization to enable interpretable and scalable protein complex identification. Code and data are available at https://github.com/hyx-1/PCIPG.

## Author summary

Proteins rarely work alone. In living cells, many proteins assemble into complexes that act as molecular machines to perform essential biological functions, such as DNA replication, RNA processing, protein degradation and cellular signaling. Identifying these complexes is therefore important for understanding how cells work and how diseases arise. However, current computational methods often rely mainly on protein–protein interaction networks, which can be incomplete or noisy, and usually ignore detailed structural information at the amino-acid residue level. In this study, we developed PCIPG, a graph-based computational framework that combines residue-level protein features, structural contact information and protein interaction networks to identify protein complexes. PCIPG learns how local structural properties of proteins are related to their interactions and complex memberships, and it can also infer missing interactions in incomplete networks. Across the evaluated yeast and human datasets, PCIPG generally achieved competitive or improved protein complex identification performance compared with the representative methods included in this study, although the relative performance varied across datasets. In addition, it highlighted biologically meaningful complex cores, candidate functional modules and residues likely to participate in protein-binding interfaces. Our results suggest that integrating structural information with interaction networks can provide a more interpretable way to study protein complex organization.

## 1 Introduction

Protein complexes are central to cellular regulation, operating as coordinated molecular machines that maintain physiological homeostasis. Fueled by rapid advances in experimental and computational methods, the structural characterization and prediction of protein assemblies has progressed substantially [1–3]. Yet a foundational question remains: which proteins intrinsically tend to assemble into complexes, especially within the large-scale proteomic maps generated by modern sequencing and proteomics technologies? This challenge—commonly termed protein complex identification—aims to determine, from a pool of proteins, the sets of subunits that co-function and co-assemble into structurally cooperative macromolecular complexes.

Existing strategies to address this problem still depend heavily on specialized experimental assays, including affinity purification, co-fractionation and cryo-electron microscopy [4–7]. In parallel, most computational approaches cast protein complex identification as module discovery in protein–protein interaction (PPI) networks, often enhanced by Gene Ontology annotations and multi-omics evidence [8–10]. By emphasizing shared cellular localization and functional similarity, these methods can recover biologically meaningful complexes at the protein level.

Despite these advances, existing computational approaches still face several major limitations. First, topology-driven methods are highly dependent on the completeness and reliability of input PPI networks. However, experimentally derived interactomes often contain both false-negative and false-positive interactions, and complex boundaries inferred solely from network connectivity can therefore be distorted by missing or noisy edges. Second, methods that incorporate Gene Ontology annotations, co-expression profiles or other omics-derived features usually operate at the protein level. Although these data sources improve functional coherence, they do not directly explain the molecular determinants that drive physical assembly. Third, conventional PPI graph representations abstract each protein as a node and each interaction as an edge, thereby discarding residue-level physicochemical properties, intra-chain structural organization and interface-related information. As a result, these methods can identify groups of associated proteins, but they usually provide limited insight into how specific subunits interact and why particular assemblies are structurally plausible.

Increasingly, however, attention is shifting to a finer resolution: pinpointing the residues and interfaces within subunits that mechanistically govern complex formation [11–13]. Current complex identification frameworks largely lack the granularity required to operate at the residue scale. For example, yeast two-hybrid (Y2H) leverages the modularity of transcription factors: binding between BAIT and PREY reconstitutes a functional transcriptional activator that drives reporter expression. As a result, Y2H reports whether two proteins interact, but not how they bind. Affinity purification coupled to mass spectrometry (AP–MS) isolates a bait protein together with co-purifying partners and identifies the components by mass spectrometry; it reveals co-complex membership, yet typically cannot discriminate direct physical contacts from associations mediated by intermediate proteins, nor localize the binding interface. Analogously, computational methods usually represent proteins as nodes and interactions as edges in a graph, an abstraction that discards the spatial organization and chemical context that reside at the residue/interface level.

The advent of AlphaFold2 has reshaped structural biology by enabling proteome-scale, high-accuracy prediction of protein monomer structures [2]. In parallel, high-throughput interaction assays, including affinity purification mass spectrometry [14] and co-fractionation mass spectrometry [15], have produced unprecedented volumes of interaction data and accelerated proteome-wide interactome mapping. Together, these advances create both an opportunity and an urgent need for computational frameworks that connect residue-level molecular determinants to network-level organization, thereby improving our mechanistic understanding of complex assembly and enhancing protein complex identification.

A desirable framework for protein complex identification should therefore satisfy three requirements. It should incorporate residue-level structural and physicochemical information to capture the local molecular basis of protein association; it should propagate such information over the PPI network to account for cellular interaction context; and it should explicitly model missing or uncertain interactions, rather than assuming that the observed interactome is complete. Meeting these requirements is particularly important for large-scale interactomes, where structural information is increasingly available but interaction evidence remains sparse, noisy and biased toward well-studied proteins.

Motivated by this gap, we introduce a residue-informed representation learning perspective that links multiple biological scales, from amino acids and protein monomers to protein complexes and interaction networks. In this view, physicochemical and structural attributes at the residue level are integrated into a learned protein representation for each protein. This residue-informed protein embedding provides a compact representation of local structural organization, residue physicochemical context and potential interface-related properties. During complex formation, these learned representations are further propagated and integrated over the PPI network, enabling PCIPG to capture higher-order organizational patterns associated with protein complex assembly.

**Fig 1:**
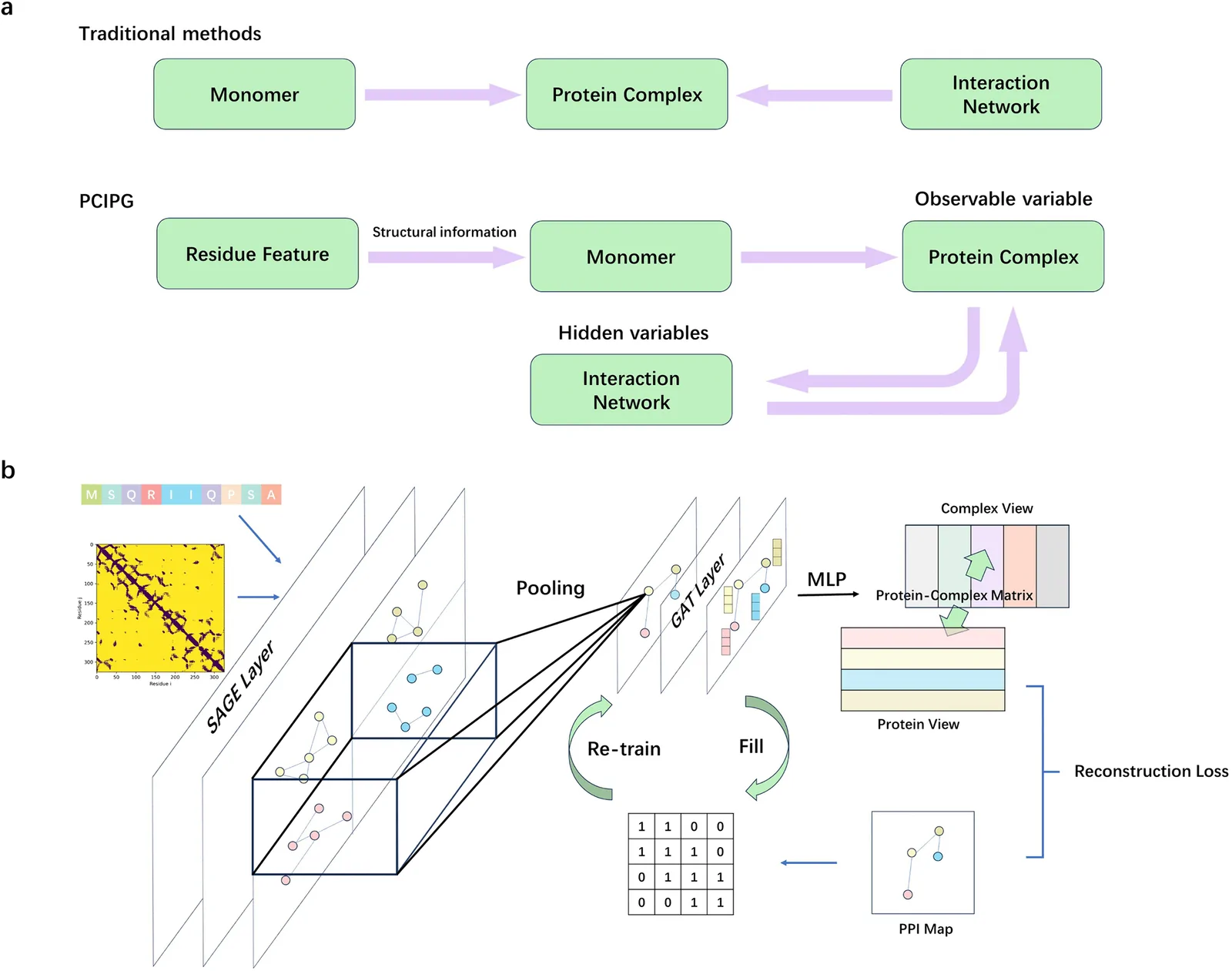
Overview of PCIPG. **(a)** Conventional protein complex identification methods mainly use protein-level functional information and PPI networks as inputs. PCIPG further incorporates residue-level physicochemical features and protein structural information. At the conceptual level, protein–complex membership is regarded as an observable biological variable, whereas the complete underlying interactome is treated as latent and incomplete because available PPI networks are partial and noisy observations of true interaction relationships. **(b)** PCIPG first encodes residue features on intra-chain contact maps using GraphSAGE. The resulting protein representations are then propagated over the PPI network using GAT.

Building on this conceptual framework, we developed PCIPG (Probabilistic Complex Identification using a Probabilistic Graph), a probabilistic graph model that connects residue-level determinants with interactome-scale organization (Fig 1). In PCIPG, we distinguish the conceptual probabilistic graphical model from the practical training implementation. At the biological modeling level, protein–complex membership is treated as an observable biological variable, because protein complexes can in principle be characterized by structural and biochemical assays [1,16,17]. By contrast, the complete underlying interaction network is modeled as a latent and incomplete variable, since available PPI maps represent noisy and partial observations of the true interactome. During training, the available PPI network is used as observed evidence, serving both as the message-passing topology and as the reconstruction target. PCIPG then infers a protein–complex membership matrix from residue-level structural features and the available PPI evidence, and couples this matrix back to the interaction network through a zero-inflated Bernoulli–Exponential reconstruction likelihood. By learning dependencies among these variables with graph neural networks, PCIPG aims to capture the cascade from residue properties and their spatial arrangement to protein-level assembly and network-level connectivity. This unified framework provides a mechanistically grounded route to protein complex identification.

Different from prior methods, PCIPG is designed as an end-to-end protein complex identification framework that integrates residue-level structural encoding, PPI-level graph propagation and probabilistic network reconstruction within a unified model. The residue-level component is introduced to construct structure-aware and residue-informed protein representations, which improves the interpretability of the predicted complexes by linking complex-level predictions to residue-level structural information. The PPI-level graph module propagates these representations over the interaction network to capture complex-related organization patterns. The probabilistic graphical formulation further couples the inferred protein–complex membership matrix with the observed interaction evidence through network reconstruction. Therefore, the novelty of PCIPG lies not in using any single component in isolation, but in combining these components within a unified probabilistic framework for accurate and interpretable protein complex identification.

Across five yeast and three human benchmark datasets, PCIPG showed strong overall protein complex identification performance relative to the representative baselines included in this study, although the magnitude and direction of the performance differences varied across datasets and metrics. PCIPG achieved favorable results on most yeast benchmark comparisons and obtained the highest F1 score on HCT116 and HEK293T among the evaluated methods. On HuRI, however, its F1 score was lower than those of AdaPPI and ClusterONE. These results indicate that PCIPG is broadly competitive across heterogeneous interaction networks, rather than uniformly superior under every evaluation setting. Functional validation via Gene Ontology (GO) enrichment indicates that the predicted complexes are biologically coherent and significantly enriched for relevant processes. In core–module mining, PCIPG reliably recapitulates established core components and further prioritizes plausible, previously unannotated candidates for follow-up investigation. In line with prior reports, complexes related to TRAPPII and PCNA loading factors recovered by PCIPG are implicated in essential cellular functions.

PCIPG further benefits from a multi-scale design that makes the identified key residues directly informative for molecular interactions. High-scoring residues prioritized by the core locus identification module showed increased correspondence with known protein-binding interfaces in the evaluated structures, suggesting that the learned residue scores contain interface-related information. In the CFTR case study, predictions generated using wild-type and ΔF508 structural inputs showed different interaction patterns and partial agreement with the corresponding experimentally profiled networks. These computational observations illustrate potential applications of PCIPG for generating residue- and network-level hypotheses. Collectively, these results establish PCIPG as an effective and interpretable framework for protein complex identification.

## 2 Results

### 2.1 Overview of PCIPG

**Fig 2:**
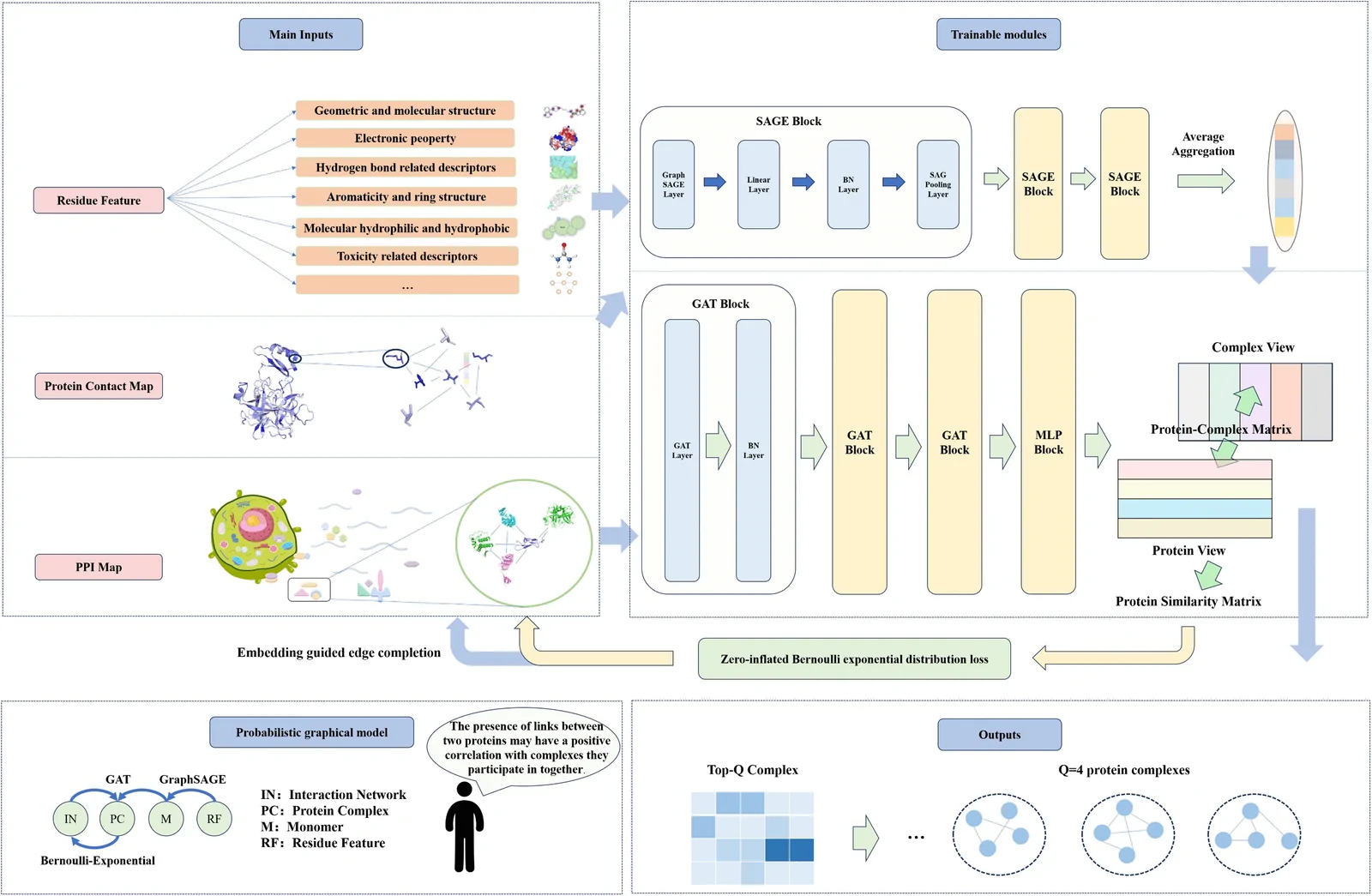
Workflow of the PCIPG framework. The framework integrates residue-level features, protein contact maps, and the PPI map as the main inputs. Residue features and contact information are first encoded by SAGE blocks to obtain protein-level representations. The PPI map is then processed by GAT blocks and an MLP block to infer protein-complex associations and protein similarity information. The model is optimized using a zero-inflated Bernoulli-exponential distribution loss. The learned embeddings further guide edge completion by estimating potential missing interactions, and the completed interaction map is used for retraining and refinement. Finally, top-Q candidate protein complexes are generated as the output.

As shown in Fig 2, PCIPG explicitly models information across four scales: residues, proteins, interactions and complexes. Residue-level physicochemical descriptors computed with RDKit are encoded into protein representations by the SAGE module using an intra-chain contact map. Each SAGE module comprises a GraphSAGE layer for residue-to-residue propagation, followed by a linear transformation with batch normalization, and a SAGPooling layer to select informative residues. Stacking three SAGE modules yields protein embeddings. These embeddings are then integrated over the protein–protein interaction network using graph attention networks (GATs). After three GAT modules, an MLP produces the protein–complex membership matrix. Finally, the interaction network is reconstructed from the membership matrix under a zero-inflated Bernoulli–Exponential model, and the model is optimized by minimizing the reconstruction loss (Eq. 26).

### 2.2 PCIPG delivers improved benchmark performance and functional coherence

**Fig 3:**
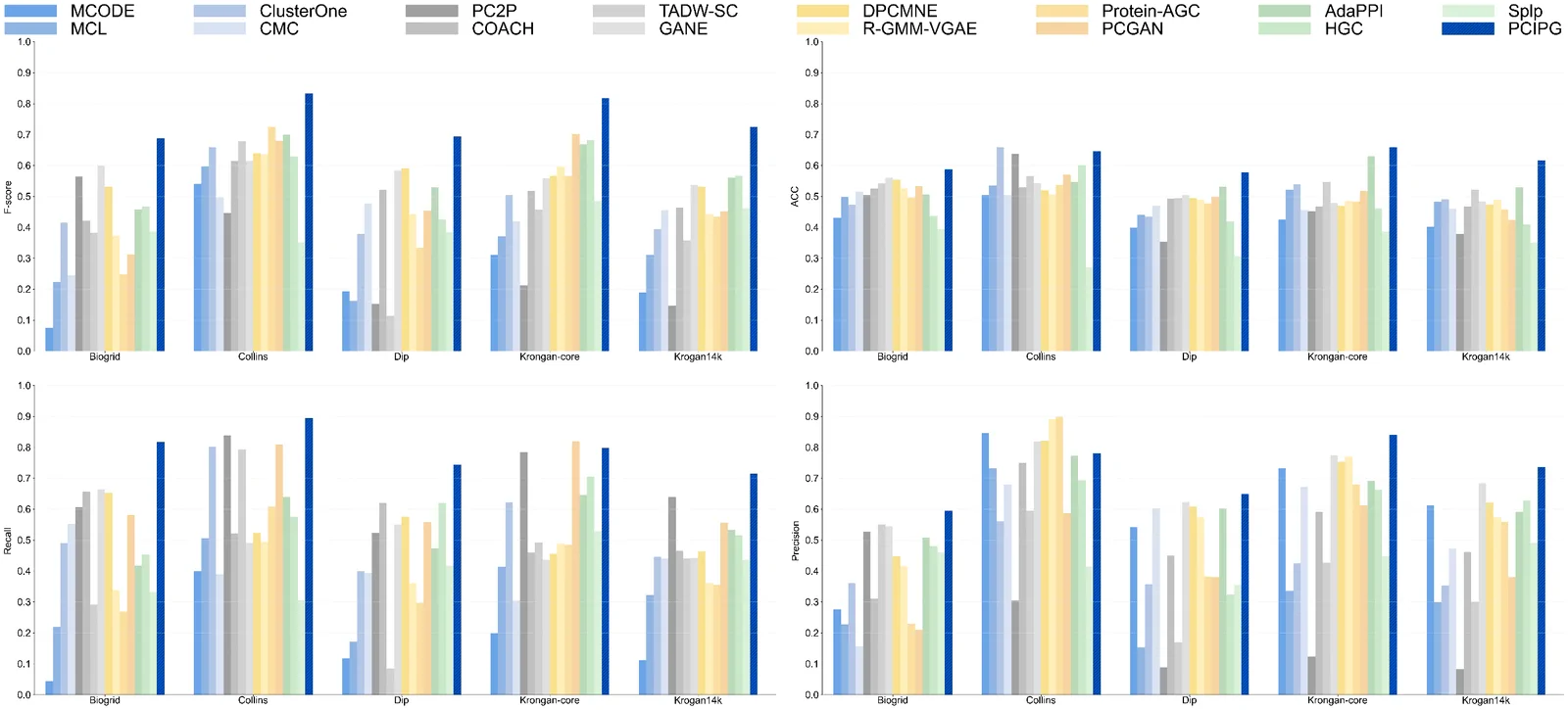
Performance comparison of protein complex detection methods on five Saccharomyces cerevisiae benchmark datasets. PCIPG achieves the strongest overall performance across datasets, with consistently higher F1 score, accuracy, recall and precision than competing methods.

To assess the effectiveness of PCIPG, we benchmarked it against 15 representative protein complex detection methods. To ensure a fair and interpretable comparison, we selected baselines from different methodological families. These methods can be broadly grouped into five categories. First, classical topology-based clustering methods, such as MCODE and MCL, identify dense or modular regions in PPI networks. Second, density-, seed- or expansion-based methods, such as ClusterONE, CMC, PC2P and COACH, search for cohesive subnetworks or expand candidate complex cores. Third, representation-learning or matrix-factorization-based methods, such as TADW-SC and GANE, learn latent protein representations from network topology and auxiliary information. Fourth, graph neural network or graph autoencoder-based methods, such as DPCMNE, R-GMM-VGAE, Protein-AGC and HGC, infer complex structures through learned graph embeddings or reconstructed network patterns. Fifth, recent deep learning or PPI-enhancement methods, such as PCGAN, AdaPPI and splp, incorporate generative modeling, adaptive PPI information or link-prediction strategies. A detailed categorization of the baseline methods is provided in Table 1. These baselines were selected because they represent both classical and recent strategies for protein complex identification and cover different assumptions about how protein complexes are organized in PPI networks. The evaluation protocol, including the normalized association-based matching between predicted and reference complexes and the definitions of Precision, Recall, F1-score, PPV, Sn and ACC, is described in detail in the Section 4. As shown in Fig 3, PCIPG achieves higher F1 score, accuracy, recall and precision than the compared baselines on most of the evaluated benchmarks, supporting its effectiveness for protein complex identification on these benchmarks.

**Table 1:** Categorization of baseline methods used for protein complex identification.

Category	Representative methods	Main principle
Topology-based clustering	MCODE, MCL	Detect dense or modular regions in PPI networks based mainly on graph topology.
Density-, seed- or expansion-based detection	ClusterONE, CMC, PC2P, COACH	Identify cohesive subnetworks, expand candidate cores or optimize local complex quality.
Representation-learning-based methods	TADW-SC, GANE	Learn latent protein representations from network topology and auxiliary information.
Graph neural network or graph autoencoder-based methods	DPCMNE, R-GMM-VGAE, Protein-AGC, HGC	Use graph neural networks, graph autoencoders or attributed graph clustering to learn complex-related network patterns.
Deep learning or PPI-enhancement methods	PCGAN, AdaPPI, splp	Incorporate generative modeling, adaptive PPI information or link-prediction strategies.

For instance, on the Collins and Krogan-core datasets, PCIPG achieves F1 scores of 0.8330 and 0.8182, respectively, which are higher than those of the competing methods included in this comparison. On the more challenging DIP and BioGRID networks, which are typically associated with lower interaction confidence and greater sparsity, PCIPG attains F1 scores of 0.6932 and 0.6879. Together, these results indicate that PCIPG maintains competitive performance across PPI networks with different noise levels and topological complexity.

Because BioGRID was used as the single development dataset for performance-informed architecture and residue-feature selection, its reported result should be interpreted as development-dataset performance rather than as a fully independent held-out estimate. After the model configuration was finalized on BioGRID, the same architecture, residue representation, training settings, and complex-generation procedure were fixed and applied to Collins, DIP, Krogan-core, and Krogan14k without further tuning against their reference complexes. These four datasets therefore provide cross-dataset evaluation of the fixed yeast model configuration.

To assess complex recognition beyond Saccharomyces cerevisiae, we evaluated PCIPG on three human datasets: HCT116, HEK293T and HuRI. As shown in Table 2, PCIPG achieved the highest F1 score among the compared methods on HCT116 and HEK293T, with values of 0.6464 and 0.5637, respectively. The corresponding strongest baseline values were 0.6336 for ClusterONE on HCT116 and 0.5403 for Protein-AGC on HEK293T. On HuRI, PCIPG achieved an F1 score of 0.4332, which was lower than AdaPPI (0.4709) and ClusterONE (0.4581), but higher than the remaining methods except these two. Thus, PCIPG performs favorably on HCT116 and HEK293T and remains competitive, although not best-performing, on HuRI. Full results for Precision, Recall and Accuracy are provided in S2 Table.

**Table 2:** F1-score comparison of PCIPG and competing methods on human datasets.

Methods	HCT116	HEK293T	HuRI
MCODE	0.4640	0.3948	0.1650
MCL	0.5148	0.3577	0.3937
ClusterONE	0.6336	0.5174	0.4581
CMC	0.5361	0.4156	0.4021
PC2P	0.6091	0.4885	0.3640
COACH	0.5593	0.4366	0.3851
TADW-SC	0.5438	0.3884	0.3610
GANE	0.5446	0.4567	0.3822
DPCMNE	0.4804	0.3448	0.2733
R-GMM-VGAE	0.4823	0.4653	0.3867
Protein-AGC	0.5593	0.5403	0.4180
PCGAN	0.5808	0.4884	0.3602
AdaPPI	0.6319	0.5280	**0.4709**
HGC	0.6080	0.5194	0.4280
splp	0.4827	0.4155	0.3520
PCIPG	**0.6464**	**0.5637**	0.4332

To assess the functional reliability of the predicted complexes, we performed Gene Ontology (GO) semantic similarity analysis on curated complexes, PCIPG-predicted complexes and randomly sampled protein sets. Random complex datasets were generated following the protocols of Qi et al. [18] and Wang et al. [19]. Briefly, we randomly sampled nodes from the PPI network to form synthetic complexes, while constraining their size distribution to follow the power-law pattern observed in the curated gold-standard complexes. The number of random complexes was fixed at five times that of the gold standard (5:1).

**Fig 4:**
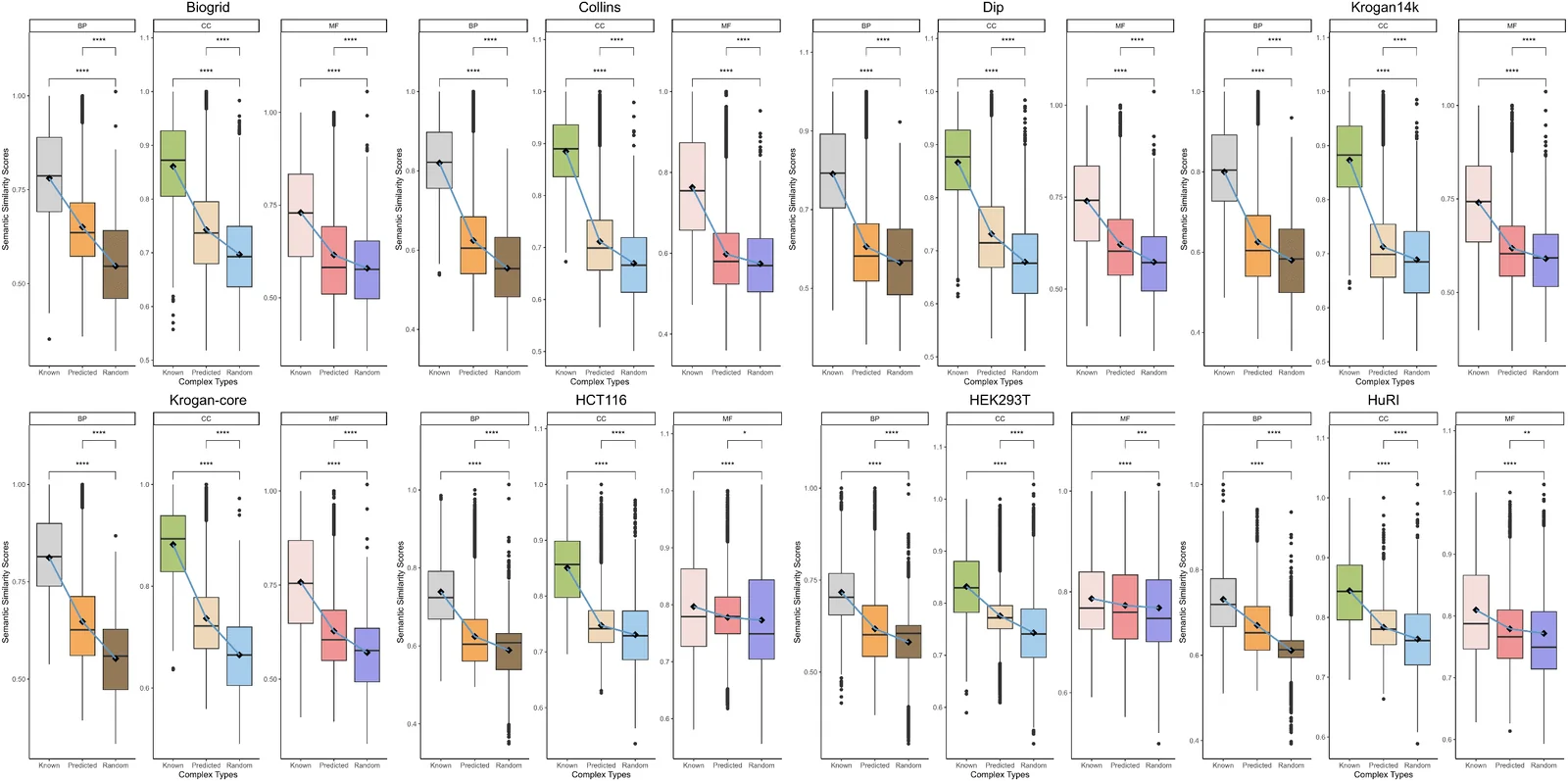
Functional coherence of complex subunits across datasets using size- and degree-matched random controls. Boxplots show the distributions of Gene Ontology semantic similarity scores for Biological Process (BP), Cellular Component (CC) and Molecular Function (MF) across BioGRID, Collins, DIP, Krogan14k, Krogan-core, HCT116, HEK293T and HuRI. Complexes are grouped as curated complexes, PCIPG-predicted complexes and size- and degree-matched random protein sets. Black points connected by lines indicate mean values. Statistical significance between groups is annotated as **** *p* < 0.0001, *** *p* < 0.001, ** *p* < 0.01, and * *p* < 0.05.

As shown in Fig 4, predicted complexes exhibit significantly higher GO semantic similarity than both size-matched random protein sets and size- and degree-matched random protein sets across Biological Process (BP), Cellular Component (CC) and Molecular Function (MF) terms. These results support the functional relatedness of subunits within the predicted assemblies and indicate that the observed functional coherence is not explained solely by complex-size distribution or node-degree effects. The consistent trends across datasets further underscore the robustness of PCIPG and highlight the functional coherence and biological plausibility of the predicted complexes.

### 2.3 Systematic ablation dissects the contributions of individual module components

**Fig 5:**
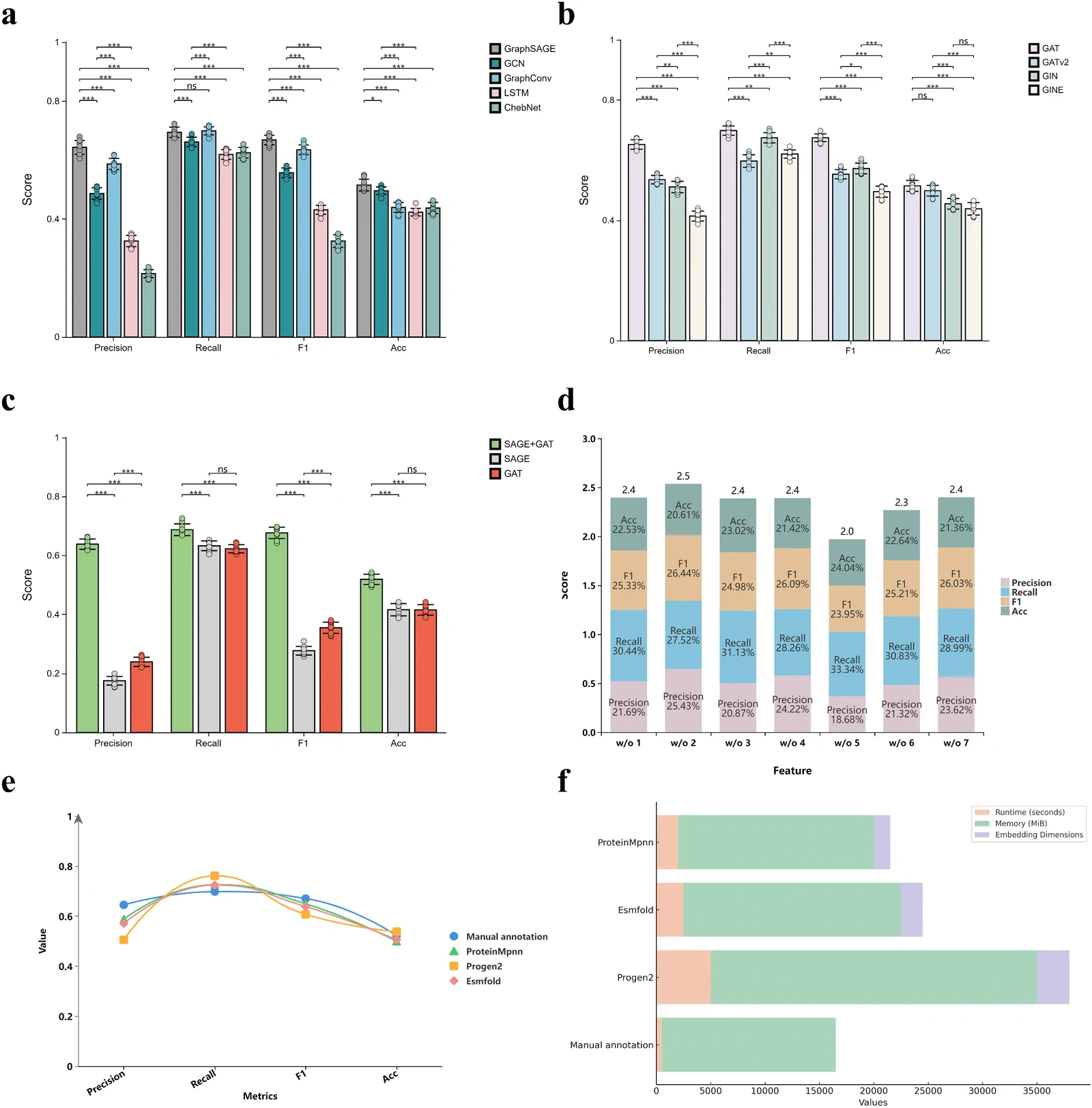
Comprehensive ablation analysis of PCIPG. **(a)** Structural feature extraction module ablation. **(b)** Interaction Network Graph Learning module ablation. **(c)** Module ablation: full model (SAGE+GAT) versus SAGE-only and GAT-only. **(d)** Residue-feature ablation by removing individual feature groups (“w/o”). The seven groups are: (1) geometric and molecular-structure descriptors; (2) electronic-property descriptors; (3) hydrogen-bond–related descriptors; (4) aromaticity and ring-structure descriptors; (5) hydrophobicity and molecular hydropathicity descriptors; (6) toxicity-related descriptors; and (7) special descriptors. **(e)** Performance comparison across different residue representation strategies. **(f)** Resource usage—including runtime, memory footprint and embedding dimensionality—across different residue representation strategies.

To systematically quantify the contribution of each component, we conducted extensive ablation studies on the yeast and human datasets, targeting the protein representation module, the protein interaction module, the residue feature extraction module and the network enhancement learning module. BioGRID was selected as the primary ablation benchmark because it is one of the largest and most widely used yeast PPI datasets in our evaluation, contains rich interaction information, and provides a challenging setting for assessing the effect of individual model components. HCT116, HEK293T and HuRI were used for network-enhancement and reconstruction-related ablations because they represent sparse human interactomes. As shown in Fig 5, Replacing GraphSAGE with alternative backbones (GCN, GraphConv, ChebNet or LSTM) showed that graph-based encoders consistently outperformed the non-graph baseline (LSTM), highlighting the importance of explicitly encoding structural topology with graph neural networks (Fig 5a). Among these variants, GraphSAGE achieved the best precision and recall, suggesting that it is particularly effective at capturing residue-level topological dependencies. Likewise, substituting the interaction module backbone (GAT) with GATv2, GIN or GINE indicated that GAT provides the most favorable trade-off between recall and accuracy (Fig 5b), consistent with its ability to model heterogeneous neighborhood contributions in networks with overlapping module.

We further assessed the necessity of the two major modules by removing them individually. Both the SAGE-only (without the interaction module) and GAT-only (without the structural representation module) variants exhibited substantial drops in F1 score and accuracy (Fig 5c), underscoring that accurate delineation of complex boundaries requires integrating residue-informed structural representations with interactome context.

We also examined the impact of residue feature design by ablating physicochemical property groups in a stepwise manner. The residue descriptor groups were initially selected according to biochemical and structural considerations. Specifically, we included residue-level properties that are known to influence protein folding, local structural organization and protein–protein interfaces, such as hydrophobicity, polarity, charge, hydrogen-bond-related descriptors, steric or volume-related properties and other physicochemical attributes. These descriptors were intended to provide complementary information about the local residue environment and the potential contribution of residues to protein interaction and complex assembly.

To examine the contribution of each residue feature group, we performed feature-group ablation analysis on the BioGRID benchmark. Each feature group was removed in turn, and the resulting model performance was compared with that of the full descriptor set. Feature groups whose removal caused clear performance degradation were retained, whereas redundant or weakly informative groups were excluded from the final representation. This procedure allowed us to balance biological interpretability, predictive performance and feature redundancy. The highest recognition accuracy was obtained when the second group was removed (Fig 5d). We believe that this reflects redundancy between the electronic-property descriptors and hydrogen-bond–related descriptors; accordingly, we excluded the former while retaining the remaining six categories in the final model. Finally, we compared manually engineered residue descriptors with embeddings from protein language models (ProGen2, ESMFold and ProteinMPNN). Although pre-trained embeddings achieved comparable—and in some cases slightly higher—predictive performance (Fig 5e), handcrafted features substantially reduced runtime, memory footprint and feature dimensionality (Fig 5f), providing a favorable balance between efficiency, interpretability and accuracy.

On the human PPI benchmarks, PCIPG performs less strongly than on Saccharomyces cerevisiae, a trend that is consistent with the substantially greater sparsity and incompleteness of currently available human interaction networks (S1 Table). To partially mitigate the impact of missing edges, we carried out network completion using protein embeddings learned by a graph attention network (GAT). Specifically, we augmented the PPI graph by adding putative interactions between protein pairs with high cosine similarity in the embedding space, thereby increasing connectivity.

To examine whether the performance gain of network enhancement comes from PCIPG-specific embedding guidance rather than generic graph densification, we compared four network settings. *No enhancement* uses the original PPI network without adding candidate edges. *Random edge addition* adds the same number of edges as PCIPG-guided enhancement by uniformly sampling unconnected protein pairs, thereby testing whether performance improves merely because the graph becomes denser. *Link-prediction-based addition* adds the same number of edges using a topology-based link prediction score (Adamic–Adar similarity [20]). This baseline tests whether local network topology alone can recover useful missing interactions. *PCIPG-guided enhancement* adds candidate interactions according to cosine similarity in the learned GAT embedding space, which integrates residue-level structural information and PPI-level interaction context. For a fair comparison, all edge-addition strategies use the same number of added edges for each dataset and are followed by the same retraining procedure.

As shown in Table 3, PCIPG-guided enhancement achieves the best performance among the compared network enhancement strategies on all three human PPI datasets. Compared with random edge addition and link-prediction-based addition, PCIPG-guided enhancement yields higher Precision, Recall, F1-score and Accuracy, suggesting that the performance gain is not merely caused by generic graph densification. Instead, the learned embedding space provides useful structure- and interaction-aware signals for prioritizing plausible missing interactions in sparse human interactomes. Additional results under alternative cosine-similarity thresholds are reported in S1 Table.

**Table 3:** Performance comparison of different network enhancement strategies on human PPI datasets.

Dataset	Network enhancement strategy	Precision	Recall	F1-score	Accuracy
HuRI	No enhancement	0.6570	0.3231	0.4332	0.4055
	Random edge addition	0.6508	0.3194	0.4285	0.4012
	Link-prediction-based addition	0.6631	0.3264	0.4375	0.4102
	**PCIPG-guided enhancement**	**0.6783**	**0.3385**	**0.4516**	**0.4240**
HCT116	No enhancement	0.8436	0.5239	0.6464	0.4740
	Random edge addition	0.8352	0.5168	0.6385	0.4675
	Link-prediction-based addition	0.8628	0.5361	0.6613	0.4896
	**PCIPG-guided enhancement**	**0.9223**	**0.5662**	**0.7016**	**0.5323**
HEK293T	No enhancement	0.7839	0.4401	0.5637	0.5079
	Random edge addition	0.7715	0.4342	0.5557	0.5013
	Link-prediction-based addition	0.8064	0.4446	0.5732	0.5111
	**PCIPG-guided enhancement**	**0.8836**	**0.4529**	**0.5989**	**0.5174**

**Fig 6:**
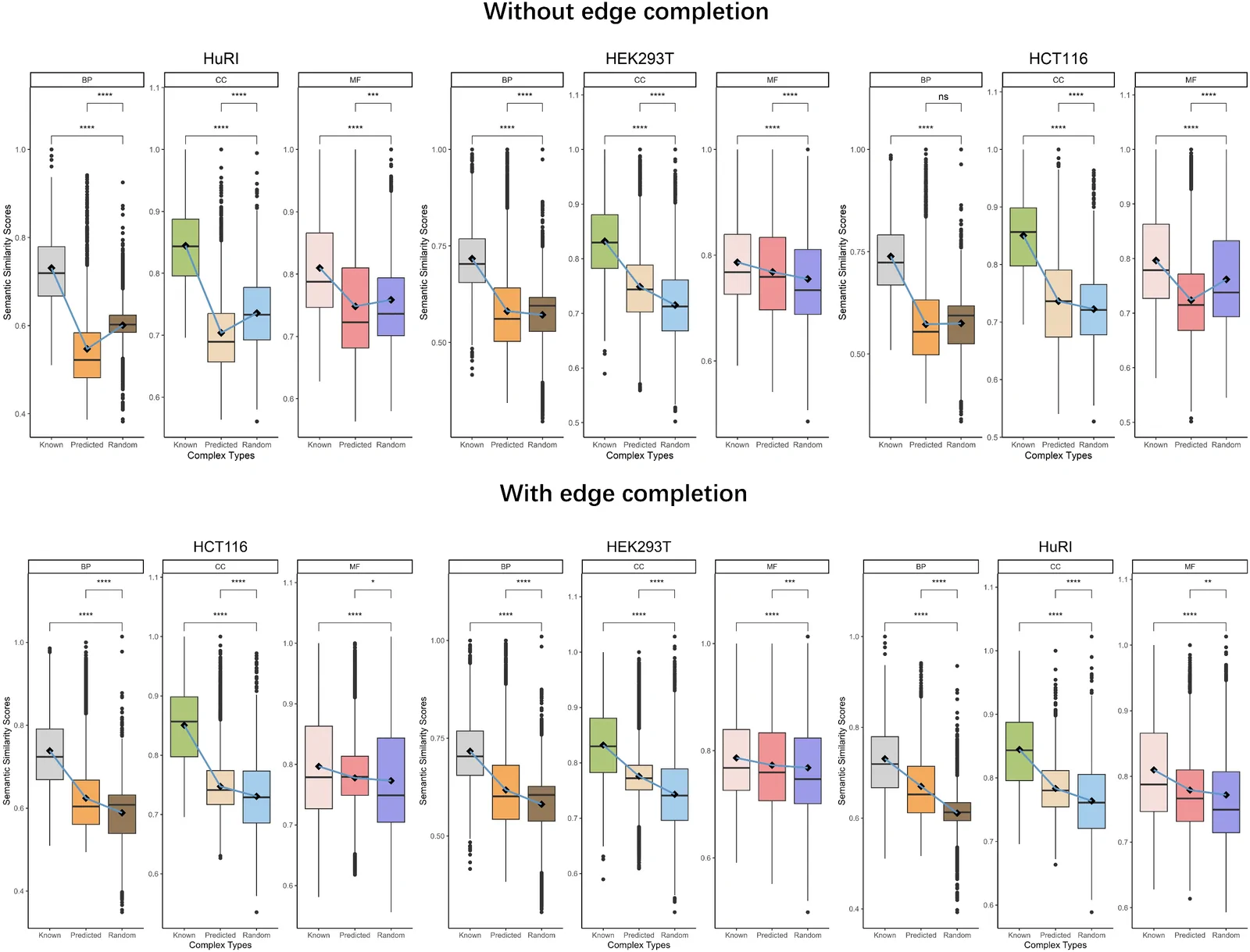
GO semantic similarity before and after embedding-guided edge completion. Boxplots show GO semantic similarity scores for Biological Process (BP), Cellular Component (CC) and Molecular Function (MF) terms in HuRI, HEK293T and HCT116. The top row shows results from the original PPI networks. The bottom row shows results after embedding-guided edge completion. Edge completion increases the GO semantic similarity of predicted complexes in the evaluated human datasets. Statistical significance was assessed using pairwise tests. Significance levels are indicated as **** *p* < 0.0001, *** *p* < 0.001, ** *p* < 0.01, * *p* < 0.05, and ns, not significant.

Consistent with the improved detection performance, GO-based functional evaluation further indicates that complexes predicted after network completion display higher functional coherence than those obtained from the original networks (Fig 6). Together, these results suggest that PCIPG can exploit latent interaction-related signals captured by network embeddings, and that moderate, embedding-guided completion offers a practical way to partially offset the incompleteness of human PPI networks and improve protein complex identification in large-scale human interactomes.

### 2.4 PCIPG recovers protein modules and core components

Early small-scale studies began to probe protein–complex organization in 2003 [21]. Building on this foundation, the landmark work by Gavin and colleagues [22] proposed the core–module–attachment model, which has since become a central paradigm in complex identification [23–25]. Using affinity purification coupled to mass spectrometry (AP–MS), this study reported 491 complexes and categorized constituent proteins into cores (present in most isoforms), attachments (present only in a subset of isoforms) and modules.

**Fig 7:**
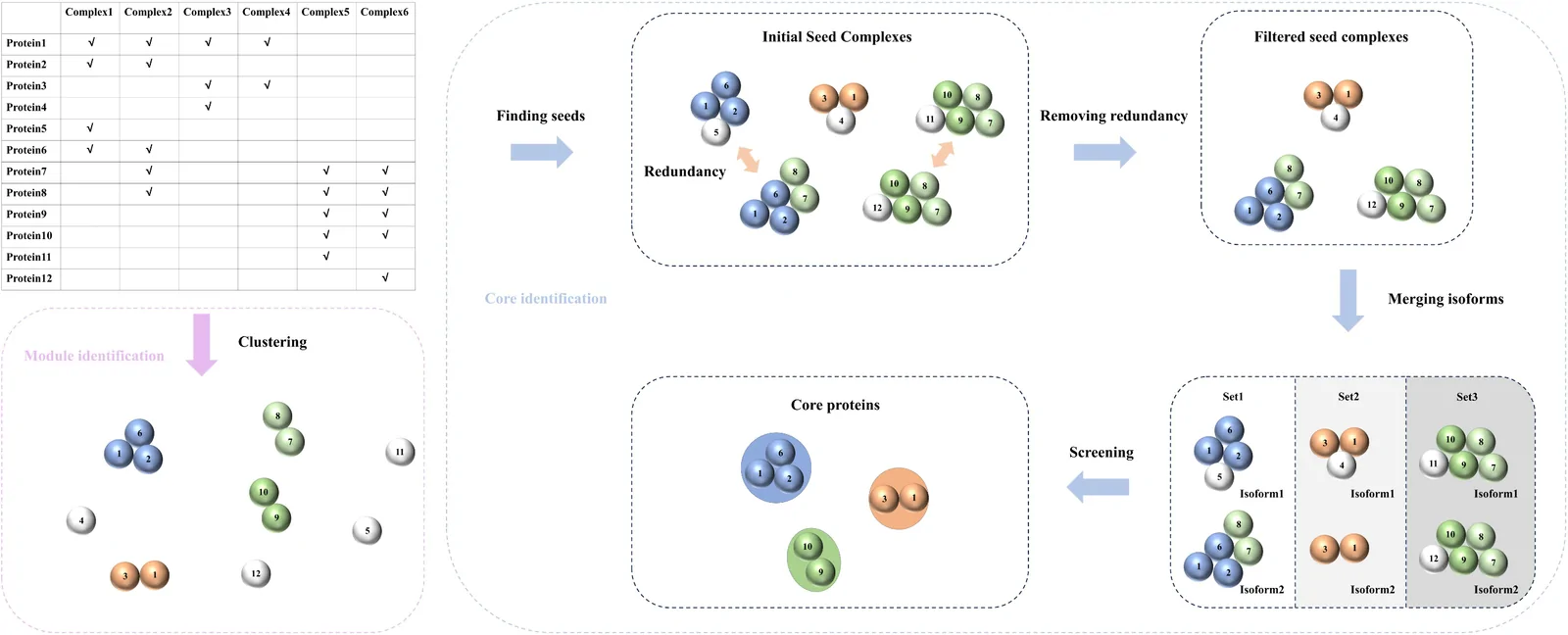
Module and core identification. Left: module identification. Each protein is represented by its membership profile in the protein–complex membership matrix *P*. Proteins with similar membership profiles are grouped by hierarchical clustering to obtain putative modules. Right: core identification. For each predicted complex *C*, we compute a complex degree $Deg(C)=\sum_{p\in C}deg(p)$, where *deg*(*p*) is the number of predicted complexes containing protein *p*. The *k* complexes with the highest *Deg* values are selected as seed complexes, where *k* denotes the number of high-degree predicted complexes retained for seed initialization. Redundant seeds are removed using the overlap score $Overlap(A,B)=|A\cap B{|}^{2}/(|A||B|)$. If *Overlap*(*A*,*B*)>*m*, the seed with the smaller *Deg* value is removed, where *m* is the overlap threshold. Remaining seeds are used to group isoform-like complexes. Core proteins are then identified according to their occurrence frequency within each group.

We adopted this catalogue as the gold standard (S3 Table) and mapped the 491 reference assemblies onto the BioGRID interaction set. Treating the GAT-derived protein-complex membership matrix *P* as a sample–feature representation, we applied hierarchical clustering to identify coherent modules (Fig 7). PCIPG predicted 306 modules, of which 187 exhibited strong overlap with experimentally defined modules; among the 122 gold-standard modules, 99 were successfully recovered. Overall, the predictions achieved NMI=0.8961, and complementary metrics (ACC=0.62, PPV=0.99) further support the reliability of the inferred modular organization.

**Fig 8:**
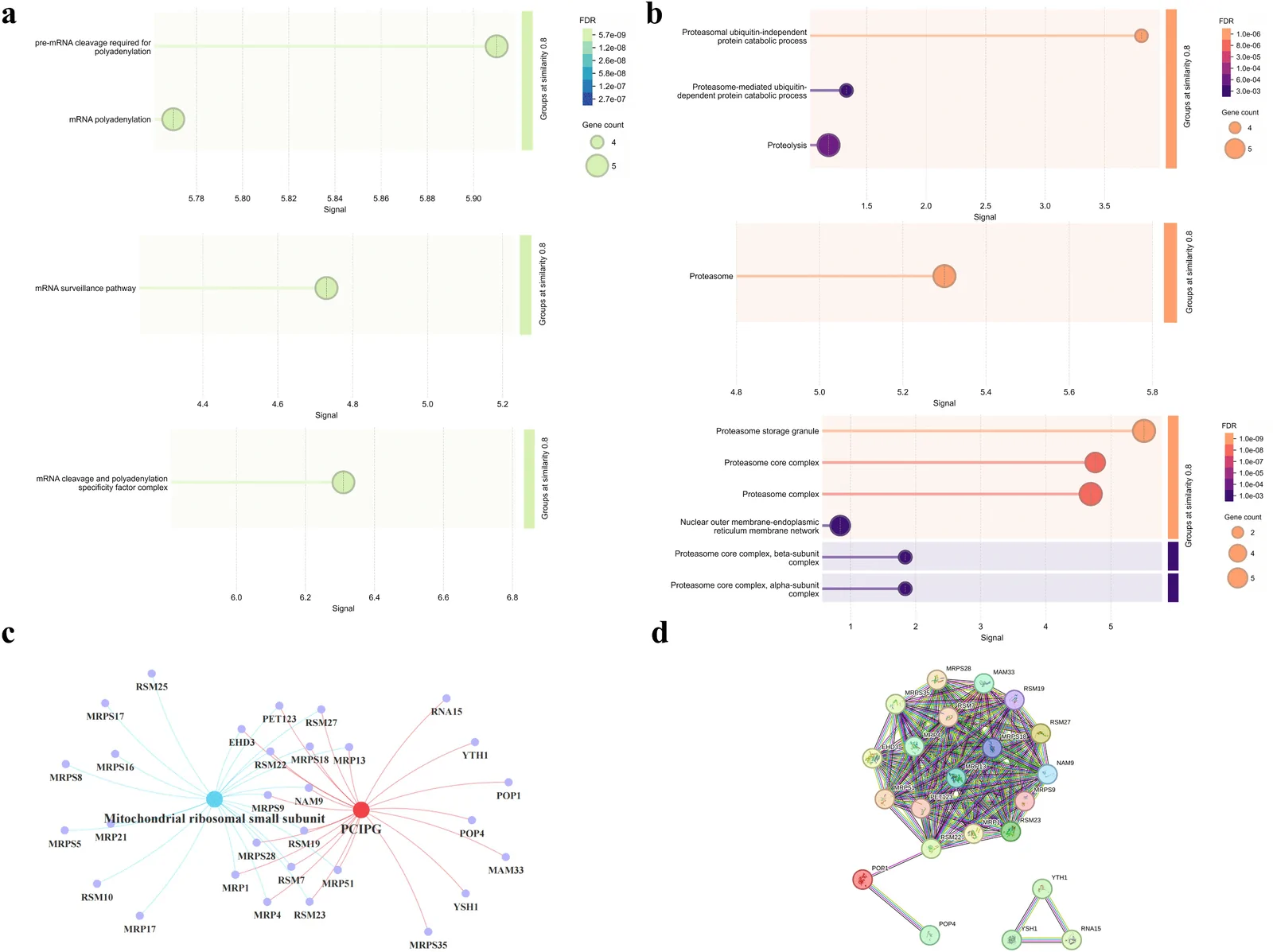
Functional enrichment and interaction evidence for predicted core proteins. **(a)** Enrichment analysis of the predicted Polyadenylation Factor I core candidates RNA15, CFT1, YSH1, YTH1 and FIP1. **(b)** Enrichment analysis of the predicted proteasome core candidates PRE2, PRE6, PRE8, PRE3 and RPN5. **(c)** Overlap between reference and predicted cores for the mitochondrial ribosomal small subunit. **(d)** STRING interaction network of high-confidence proteins in the mitochondrial small-subunit cluster. Edge thickness indicates interaction confidence.

To identify core components, PCIPG first clustered isoform-like assemblies and then applied a frequency-based two-sample Welch’s *t*-test to detect stable subunits within each cluster (Fig 7). For each protein, the null hypothesis was that its mean occurrence frequency in the target cluster equalled its mean frequency across all other clusters, whereas the alternative hypothesis was that the protein occurred more frequently in the target cluster than elsewhere. Among the five most significant hits—RNA15, CFT1, YSH1, YTH1 and FIP1—four (CFT1, YSH1, YTH1 and FIP1) are established core subunits of Polyadenylation Factor I, whereas RNA15, although not annotated as a core component, resides within the same module. KEGG/GO enrichment further indicated that all five proteins are involved in mRNA surveillance and pre-mRNA cleavage processes required for polyadenylation (Fig 8a), underscoring their coordinated roles. In another predicted set (PRE2, PRE6, PRE8, PRE3 and RPN5), four proteins (PRE2, PRE6, PRE8 and PRE3) are components of the 20S proteasome core particle, and enrichment analysis linked all five proteins to proteasome storage granules and proteolysis (Fig 8b).

We additionally reported all high-frequency proteins with confidence greater than 0.95. For the mitochondrial ribosomal small subunit, PCIPG recovered 15 of 23 reference core proteins among 22 predicted candidates (Fig 8c). Notably, one predicted protein, MRPS35—currently not classified as a core subunit—exhibited dense and functionally coherent interactions with most established core proteins of the mitochondrial ribosomal small subunit (Fig 8d), nominating it as a promising candidate core component.

Together, these results indicate that PCIPG can recover known core subunits and nominate previously unrecognized candidates, providing actionable hypotheses for experimental validation.

### 2.5 PCIPG identifies complexes implicated in essential biological processes

**Fig 9:**
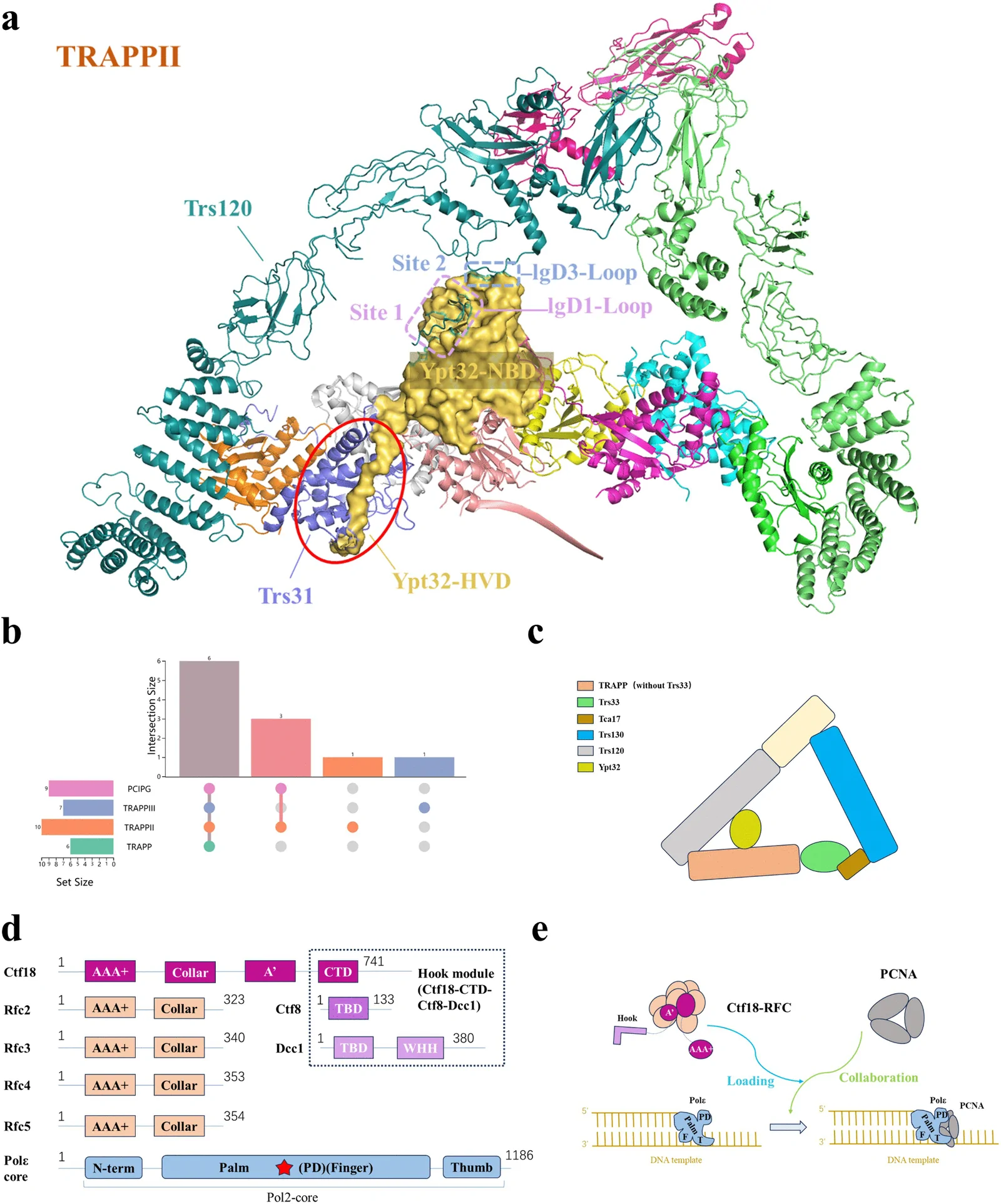
Representative PCIPG-identified complexes involved in essential biological processes. **(a)** Structural view of TRAPPII bound to Ypt32, highlighting interfaces involving Trs120, Trs31 and Ypt32. The organization of the experimentally reported complex was redrawn and adapted from [31]. **(b)** Overlap between the PCIPG-predicted complex and annotated TRAPP-family assemblies. **(c)** PCIPG identifies Trs120, a TRAPPII-specific subunit with an important functional role. **(d)** Ctf18, Ctf8 and Dcc1 form an alternative PCNA loader together with RFC family subunits. **(e)** Schematic of PCNA loading and DNA polymerase collaboration during DNA replication.

We next examined whether complexes recovered by PCIPG correspond to functionally specific assemblies implicated in essential biological processes. One illustrative example is the TRAPP complex, a multi-subunit tethering complex that exists in three forms—core TRAPP/TRAPPI, TRAPPII and TRAPPIII—and functions in vesicle trafficking and membrane fusion. All three share a conserved six-subunit core (Bet3, Bet5, Trs20, Trs23, Trs31 and Trs33), which constitutes the core TRAPP/TRAPPI complex [26]. TRAPPII incorporates four additional subunits (Trs120, Trs130, Trs65 and Tca17), whereas TRAPPIII contains the distinct subunit Trs85 [27]. In yeast, TRAPPII acts as a guanine nucleotide exchange factor (GEF) for Ypt31/32, promoting the maturation of post-Golgi vesicles from cisternal precursors [28]. Structural studies further revealed that TRAPPII forms a three-layered rhomboid-shaped dimer, with two TRAPPI cores constituting the outer layers and TRAPPII-specific subunits occupying the central layer [29]. In the absence of Ypt32, TRAPPII samples both “open” and “closed” conformations; Ypt32 binding stabilizes the “closed” state, which creates a favourable environment for nucleotide exchange, with Trs120 playing a central role. Specifically, two Trs120 domains (IgD1 and IgD3) contact Ypt32 to promote the required rearrangements (Fig 9a), and interactions between Trs120 and the TRAPP core further enhance nucleotide release [30].

Using PCIPG, we identified a nine-subunit complex comprising Bet3, Bet5, Trs20, Trs23, Trs31, Trs33, Trs120, Trs130 and Trs65. All six TRAPP core components were recovered (Fig 9b). Moreover, PCIPG identified three of the four TRAPPII-specific subunits, corresponding to a 75% recovery rate. Notably, the prediction includes Trs120, a key determinant of functional divergence between TRAPP and TRAPPII (Fig 9c) and an essential regulator of late-Golgi trafficking [31]. Together, these results indicate that PCIPG can recover subunits of high functional significance within experimentally characterized assemblies.

A second example concerns the proliferating cell nuclear antigen (PCNA) loader. DNA polymerases—including leading-strand Polϵ and lagging-strand Polδ—depend on the PCNA sliding clamp to achieve high activity and processivity during replication. Because PCNA forms a closed ring, it must be transiently opened and reclosed around DNA by an ATP-dependent clamp loader (Fig 9e). Classical studies established that replication factor C (RFC), a pentameric ATPase composed of Rfc1–Rfc5, mediates this process on both leading and lagging strands [32,33].In Ctf18-RFC complex, Ctf18 subunit replaces Rfc1 of RFC complex. So the main ATPase module is formed by Ctf18-Rfc2–5. Then, the Ctf18 subunit C-terminal tail together with Dcc1 and Ctf8 form a separate module. Notably, recent structural and biochemical evidence has confirmed that Ctf18-RFC specifically loads PCNA onto the leading strand [34] (Fig 9d), thereby validating our computational finding. Collectively, these case studies underscore PCIPG’s ability not only to recover canonical assemblies but also to anticipate alternative architectures with specialized biological roles.

**Fig 10:**
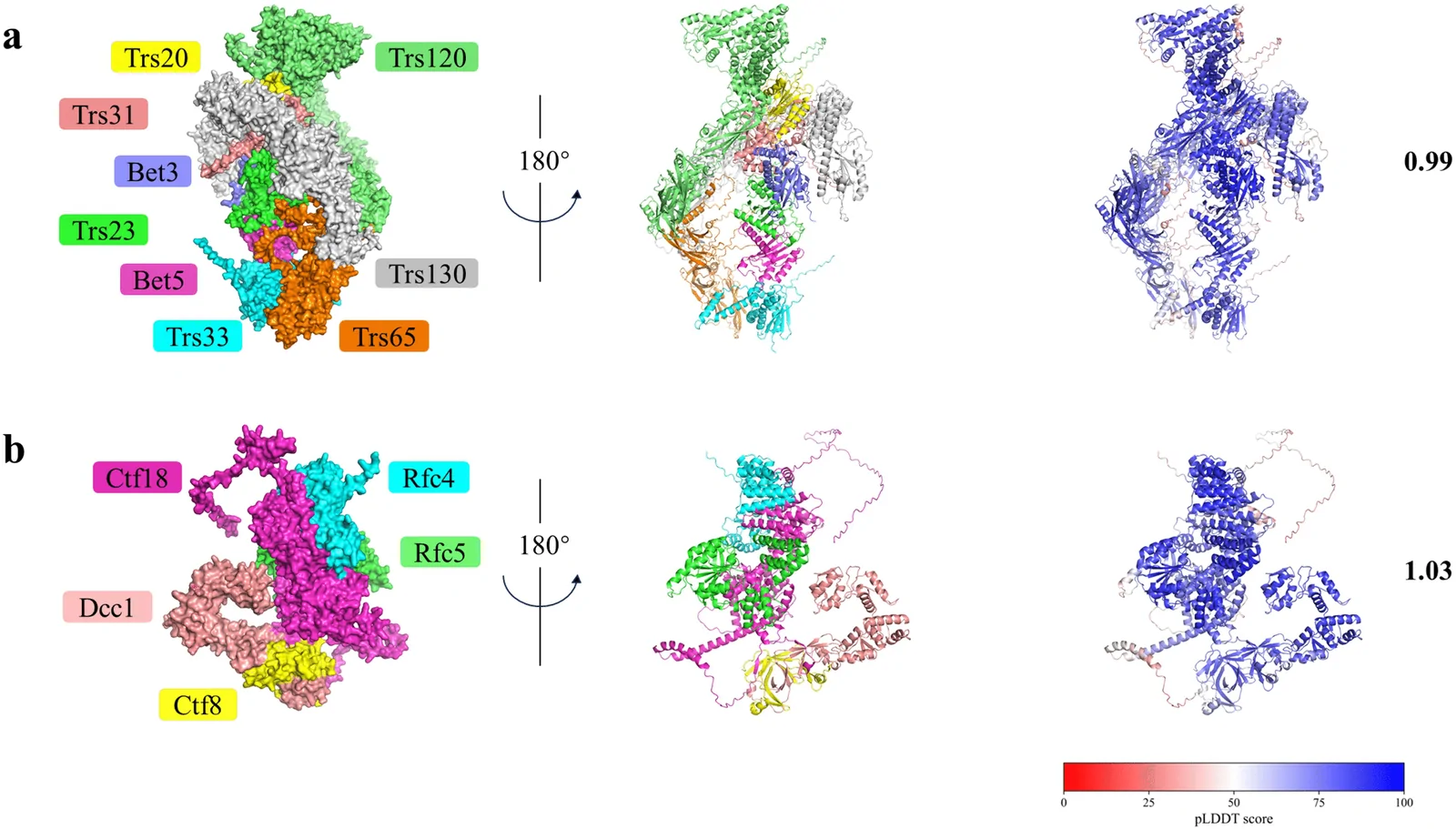
AlphaFold3 structural models of representative PCIPG-predicted complexes. **(a)** Predicted structure of the TRAPPII-like complex containing Bet3, Bet5, Trs20, Trs23, Trs31, Trs33, Trs120, Trs130 and Trs65. **(b)** Predicted structure of the Ctf18-RFC complex containing Ctf18, Dcc1, Ctf8, Rfc4 and Rfc5. In each panel, the rightmost structure is colored by pLDDT confidence score. The IPTM+PTM scores are shown to indicate the structural confidence of the predicted assemblies.

We further leveraged AlphaFold3 to model the structures of the predicted TRAPPII-like complex (Fig 10a) and the Ctf–Rfc complex (Fig 10b), and assessed their structural plausibility using the IPTM+PTM confidence metric [35,36]. Both assemblies received high scores (0.99 and 1.03, respectively), consistent with stable and biologically plausible complex formation. Together, these analyses support that PCIPG recovers complexes that are not only functionally relevant but also structurally coherent.

### 2.6 Core locus recognition module highlights residues associated with interfaces and conservation signatures

We assessed PCIPG’s ability to predict interfacial residues using experimentally resolved complexes 8EKI, 6YGD, 6VZG, 9GD4 and 5L52, which contain proteins from the Collins dataset. Complex 5L52 was additionally used as a representative case for multi-protein assemblies. Two residues were considered in contact if the Euclidean distance between their cross-chain $C_{\alpha }$ atoms was less than 8 Å, and prediction performance was quantified by precision.

**Fig 11:**
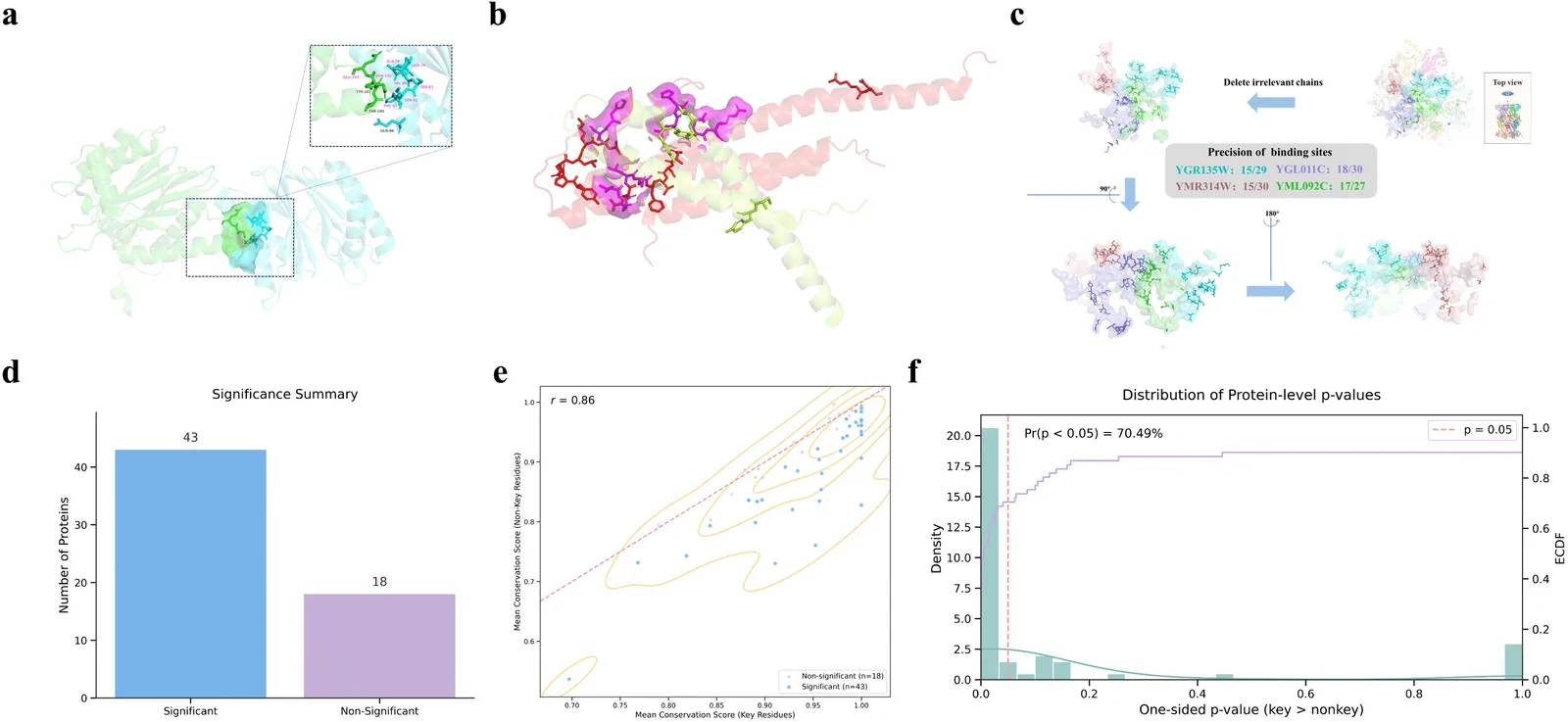
Interface-residue prediction and conservation analysis. **(a–c)** Examples of PCIPG-predicted key residues at protein–protein interfaces. Interacting regions are shown as molecular surfaces, and predicted key residues are highlighted as sticks. **(d)** Number of proteins whose predicted key residues show significantly higher conservation than non-key residues. **(e)** Mean conservation scores of key and non-key residues for each protein. **(f)** Distribution of one-sided protein-level *p*-values for the test that key residues are more conserved than non-key residues.

For binary protein–protein interactions, binding sites were inferred by applying Steiner-forest graph optimization (Eq. 29) to the residue candidates selected by SAGE pooling (S5 Table). Compared with three established methods—DeepPPISP [11], PGT [12] and HN-PPISP [13]—PCIPG achieved the highest precision on 75% of the tested targets (Table 4). Representative examples include the YDR498C–YGL098W and YGR135W–YER012W pairs (Fig 11a,11b).

**Table 4:** Comparison of interface-residue prediction precision (%) for PCIPG and baseline methods across representative protein complexes.

PDB ID	Protein (Gene)	PCIPG	DeepPPISP	PGT	HN-PPISP
8EKI	YDR498C	**31.58**	20.93	28.49	17.20
	YGL098W	**44.44**	26.37	37.55	25.19
6YGD	YPR051W	**53.33**	17.25	40.82	32.54
	YEL053C	**12.68**	11.04	5.07	7.85
6VZG	YPR034W	0.00	15.86	**26.93**	19.24
	YGR275W	**35.00**	28.07	23.41	31.22
9GD4	YLR314C	**25.00**	12.01	22.38	16.08
	YHR107C	**20.00**	15.78	14.07	13.25
5L52	YGL011C	**23.08**	14.88	19.63	5.04
	YML092C	**22.22**	19.51	13.49	8.81
5L52	YGR135W	8.70	24.33	21.03	**34.90**
	YER012W	**26.09**	5.59	18.32	17.23
5L52	YGR135W	7.69	**20.04**	3.71	11.62
	YML092C	**37.04**	29.52	25.30	33.57
5L52	YGR135W	7.41	9.63	22.00	**25.07**
	YER094C	**19.23**	12.44	15.08	8.90

For multi-protein assemblies, candidate interfacial residues were directly taken from the outputs of the SAGE pooling layer (Eq. 12). For 5L52, PCIPG achieved precision exceeding 50% for YGL011C, YML092C, YGR135W and YMR314W, highlighting its ability to capture interfacial determinants in multimeric contexts (Fig 11c). Collectively, these results indicate that by integrating information across scales, PCIPG learns interaction dependencies spanning multiple proteins during training, which may contribute to more accurate identification of protein–protein interfaces.

We also examined the extent to which residues highlighted by PCIPG as key—those retained by the final SAGE pooling layer—are conserved across evolution. Using the Collins dataset, we computed residue-level conservation scores from multiple sequence alignments (Eq. 31). After excluding proteins whose residues exhibited near-uniform conservation, we compared key and non-key positions within each protein. As shown in Fig 11d–11f, key residues consistently display higher conservation than non-key residues.

At the global level, a one-sided two-sample *t*-test confirmed significantly elevated conservation scores for key residues ($p=8.2256\times 10^{-43}$). At the single-protein level, 43 proteins showed significant differences whereas 18 did not, indicating that the trend holds broadly across the proteome (Fig 11d). A scatterplot of per-protein mean conservation scores revealed a strong correlation (*r* = 0.86) between key and non-key positions, with key residues systematically shifted upward (Fig 11e). Moreover, the distribution of one-sided *p*-values was strongly skewed toward zero (Fig 11f), with a pronounced enrichment of proteins in the low-value regime, suggesting that the conservation advantage is widespread rather than driven by a small number of outliers.

Together, these results suggest that by integrating protein structural context with residue physicochemical properties, the high-scoring residues selected by the SAGE pooling module capture evolutionarily constrained positions, consistent with their potential functional importance and supporting the utility of SAGE for protein characterization.

### 2.7 Computational analysis of mutation-associated differences in the inferred CFTR interaction landscape

To further evaluate PCIPG’s ability to model how single-protein mutations reshape interaction networks, we applied it to characterize mutation-specific interaction landscapes. We used a dataset from Ref. [22], which experimentally profiled the protein–protein interaction (PPI) networks of the ΔF508 CFTR mutant and wild-type (WT) CFTR. The mutant and WT networks contain 951 and 759 interactions, respectively, with 511 edges shared between them (S4 Table). These interactions include both direct and indirect CFTR partners.

We trained PCIPG using CFTR structures before and after mutation and adopted a confidence threshold of η=0.95 to focus on high-confidence interactions. We first extracted the 511 interactions shared by WT and mutant networks and treated them as mutation-stable edges. Using the WT CFTR structure together with these 511 stable interactions, PCIPG inferred an additional set of 149 interactions that are absent from the shared set but present in the experimentally determined WT network (759 edges), demonstrating PCIPG’s ability to recover the wild-type CFTR interaction network. We then collected the 128 proteins involved in these 149 interactions and retrieved their interactions within the shared network, combining them with the newly inferred edges to form an expanded WT interaction landscape.

**Fig 12:**
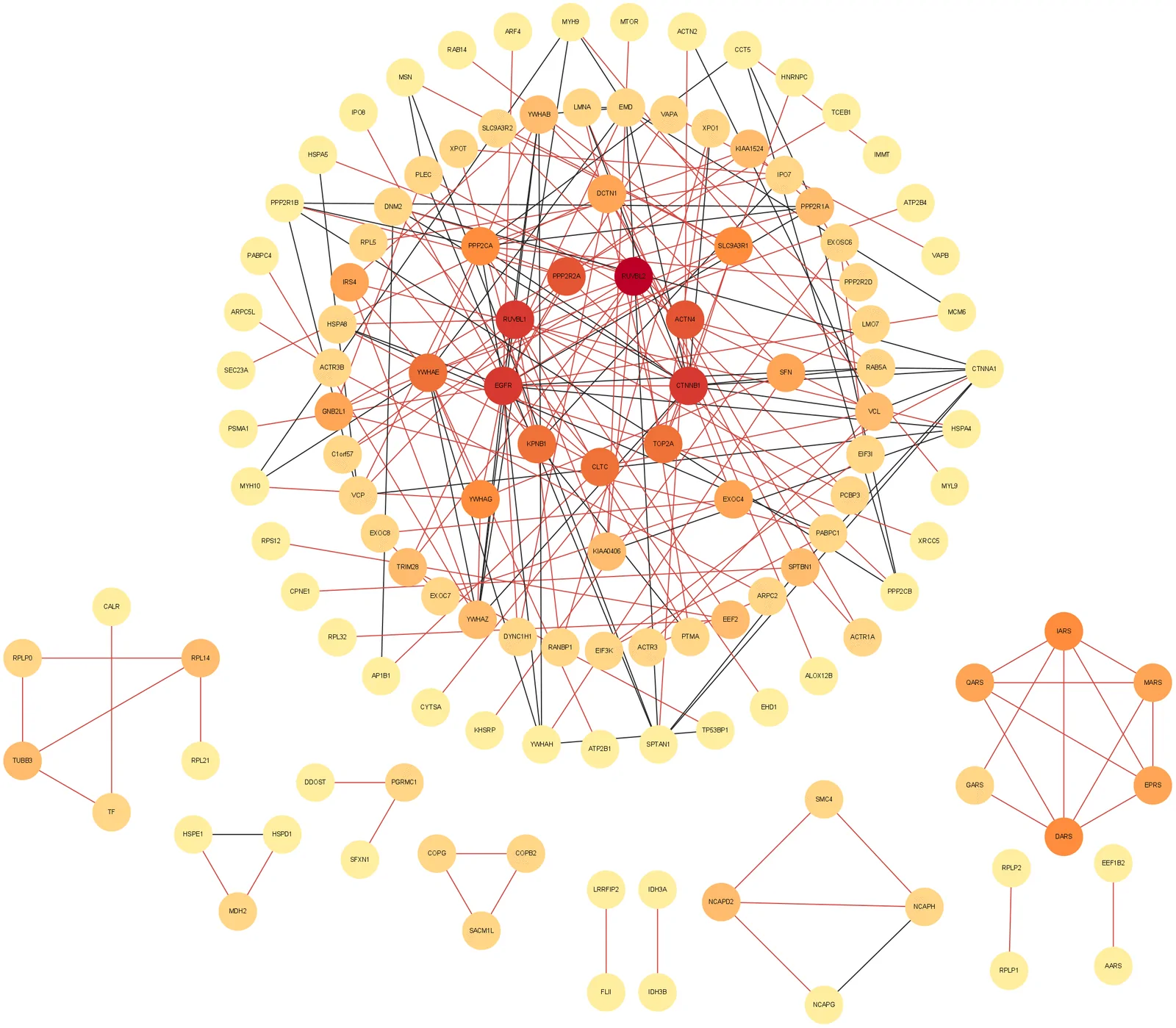
Wild-type interaction network (native state). The network depicts the interaction landscape associated with wild-type (WT) CFTR, with nodes representing CFTR-associated proteins and edges denoting protein–protein interactions in the native condition. Black edges indicate interactions shared between WT and ΔF508 CFTR, whereas red edges represent WT-specific interactions successfully recovered by PCIPG.

**Fig 13:**
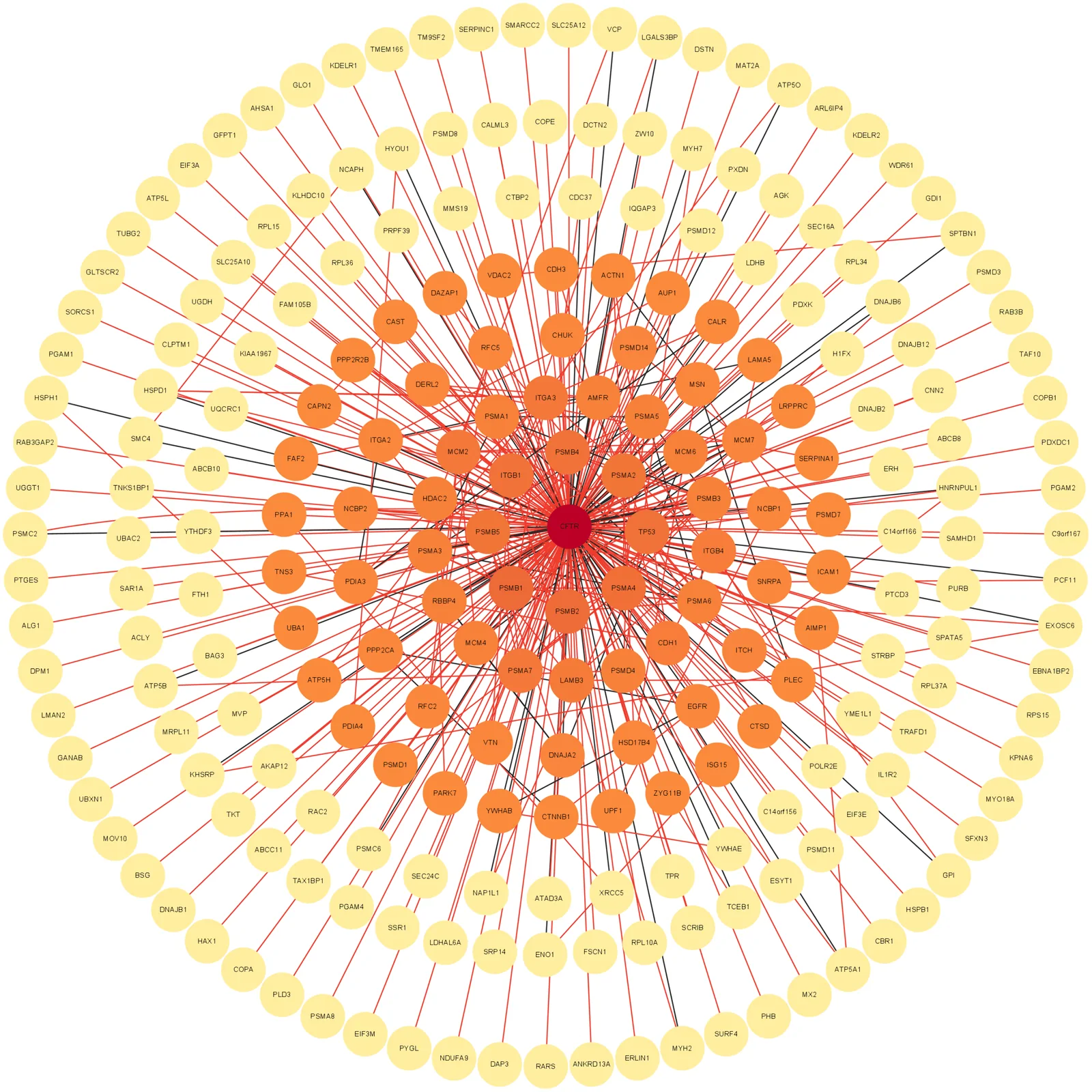
Interaction network in the mutant state. The network depicts the interaction landscape associated with ΔF508 CFTR, with nodes representing CFTR-associated proteins and edges denoting protein–protein interactions under the mutant condition. Black edges indicate interactions shared between mutant and wild-type CFTR, whereas red edges represent mutation-specific interactions successfully recovered by PCIPG.

As shown in Fig 12, black edges denote interactions from the shared (mutation-stable) set, whereas red edges indicate interactions newly recovered by PCIPG. Notably, the 128 proteins exhibit distinct clustering patterns in the shared versus WT-specific networks. In addition to preserving the core interaction backbone, PCIPG substantially recovers the wild-type interaction network, including peripheral and locally connected sub-networks, highlighting its ability to reconstruct fine-grained interaction structure. Similarly, we used the mutant CFTR structure together with the 511 mutation-stable interactions to infer the disease-state network. PCIPG recovered 309 additional interactions that were absent from the shared set yet present in the experimentally determined mutant network (951 edges). The subnetwork induced by these 309 interactions involved 232 proteins. We then examined the network of these proteins in the stable interaction set and combined it with the newly recovered edges for comparison (Fig 13). PCIPG delineated an interaction landscape centered on mutant CFTR, demonstrating its ability to recover the mutant-specific interaction network by leveraging mutation-induced structural changes to resolve differential interaction patterns relative to the WT state.

The inferred wild-type and mutant interaction sets differed, with an overall Jaccard-based difference of 0.112 under the analysis defined in Eq. 35. This observation suggests that PCIPG can produce distinguishable interaction predictions when supplied with different CFTR structural states. The agreement of subsets of these predictions with the experimentally profiled networks supports their potential biological relevance.

## 3 Discussion

PCIPG provides a multi-scale perspective for protein complex identification by connecting residue-level structural cues with interactome-level organization. Its contribution does not depend on claiming novelty for the individual graph-learning modules. Instead, it lies in organizing these established components into a unified framework. Unlike conventional PPI topology-based methods that mainly search for dense or cohesive subnetworks, PCIPG attempts to model how physicochemical and structural properties of residues are integrated into protein representations, how these representations propagate through the interaction network, and how protein-complex memberships can be inferred under incomplete and noisy interaction evidence. This design has two implications. Methodologically, it suggests that protein complex identification can benefit from explicitly coupling molecular-scale structural information with network-scale graph learning. Biologically, it provides a route to generate more interpretable hypotheses, because predicted complexes can be further examined in terms of core modules, interface-related residues, structural plausibility and perturbation-associated interaction rewiring.

### 3.1 Zero-inflated Bernoulli–Exponential modeling captures protein interaction strength distributions

**Fig 14:**
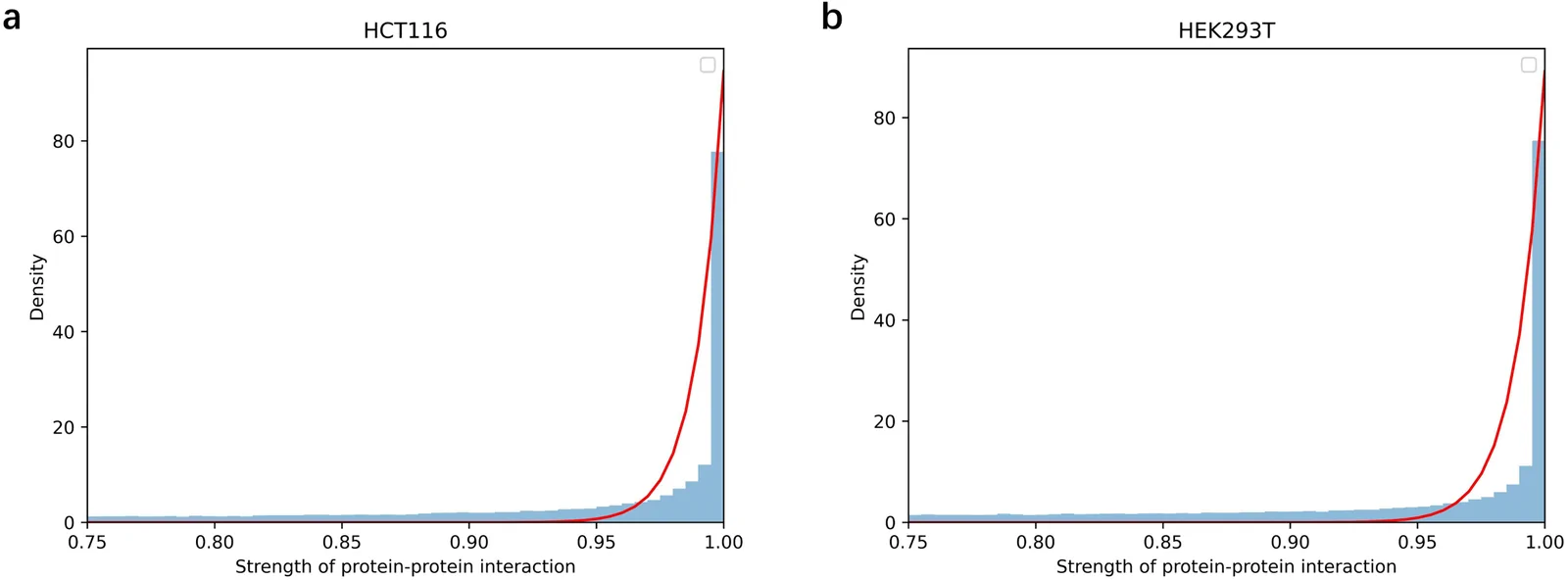
Empirical distributions of interaction strengths in the HCT116 and HEK293T datasets. Histograms show the observed edge-weight distributions, and solid curves denote the fitted exponential probability density for non-zero interaction strengths.

To assess the suitability of the proposed zero-inflated Bernoulli–Exponential model for weighted PPI data, we fit the model to two human interaction datasets with quantitative edge strengths, HCT116 and HEK293T. As shown in Fig 14, the (transformed) exponential component closely matches the distribution of non-zero interaction strengths, whereas the Bernoulli component naturally captures the excess zeros.

We further performed an ablation analysis by (i) using a Bernoulli-only model (the degenerate case of the Bernoulli–Exponential formulation) and (ii) using cosine similarity between protein pairs in the predicted protein-complex membership matrix as a proxy interaction score, and compared these variants with the full PCIPG model. As summarized in Table 5, the zero-inflated Bernoulli–Exponential formulation yields the strongest complex identification performance, supporting its effectiveness in coupling protein-complex membership patterns to observed interaction strengths.

**Table 5:** Performance comparison of alternative protein–protein interaction network reconstruction strategies.

	Approach	Precision	Recall	F1-score	Accuracy
HCT116	Cos-sim	0.7324	0.4507	0.5580	0.4318
	PCIPG(only-Bou)	0.7676	0.4783	0.5894	0.4691
	PCIPG	**0.8436**	**0.5239**	**0.6464**	**0.4740**
HEK293T	Cos-sim	0.7295	0.3912	0.5093	0.4580
	PCIPG(only-Bou)	0.7452	0.4104	0.5293	0.4467
	PCIPG	**0.7839**	**0.4401**	**0.5637**	**0.5079**

The empirical distribution analysis further supports the use of the zero-inflated Bernoulli–Exponential model. In HCT116 and HEK293T, the Bernoulli component naturally accounts for the excess zero entries in the PPI matrix, whereas the transformed Exponential component captures the right-skewed distribution of non-zero interaction strengths. Moreover, the ablation comparison shows that the full Bernoulli–Exponential formulation outperforms both the Bernoulli-only variant and the cosine-similarity reconstruction baseline, indicating that modeling both interaction occurrence and non-zero interaction strength provides a more informative reconstruction objective for protein complex identification.

### 3.2 PCIPG maintains robust complex identification under network and structural perturbations

Although experimentally determined structures are available for only a subset of proteins, most lack high-confidence resolved 3D conformations; consequently, structure-aware analyses often rely on in silico predictions. To examine whether PCIPG depends critically on experimentally solved structures, we performed a control experiment in which native structures were replaced with AlphaFold-predicted models for all proteins across the five Saccharomyces cerevisiae datasets. As shown in Table 6, this substitution leads to only marginal changes in PCIPG performance. These results suggest that PCIPG is largely insensitive to the provenance and uncertainty of structural inputs, supporting its applicability in settings where only predicted structures are available.

**Table 6:** Performance comparison using predicted versus native structures on five Saccharomyces cerevisiae PPI networks.

	Structure	Precision	Recall	F1-score	Accuracy
Biogrid	Predicted	0.5631	0.8005	0.6611	0.5724
	Native	0.5948	0.8155	0.6879	0.5880
Collins	Predicted	0.7519	0.8840	0.8126	0.6437
	Native	0.7799	0.8939	0.8330	0.6461
Dip	Predicted	0.6125	0.7501	0.6744	0.5429
	Native	0.6489	0.7440	0.6932	0.5776
Krogan-core	Predicted	0.8386	0.7724	0.8041	0.6270
	Native	0.8407	0.7969	0.8182	0.6585
Krogan14k	Predicted	0.6904	0.7026	0.6964	0.6138
	Native	0.7359	0.7139	0.7247	0.6155

We assessed the robustness of PCIPG to perturbations in protein–protein interaction networks across five Saccharomyces cerevisiae datasets. Specifically, we randomly removed 5%, 10% or 20% of the true interaction edges and, independently, added 5%, 10% or 20% false-positive edges by sampling protein pairs without known interactions. All other training and inference settings, including hyperparameters, were kept identical. Performance was summarized by the F1 score and reported relative to the unperturbed network (Origin).

As shown in Table 7, PCIPG remains resilient to 5–20% random edge deletions or additions. In most settings, performance changes relative to Origin are modest, and slight improvements are observed in roughly half of the perturbation conditions (for example, on Collins and Krogan-core, and on DIP under edge additions of ≥10%). We suggest two contributing factors. First, PCIPG integrates residue physicochemical properties and structure-informed protein representations, which may buffer the impact of spurious or missing edges on complex delineation. Second, mild random perturbations can act as a form of noise regularization, reducing overfitting and promoting smoother, more coherent functional module boundaries.

**Table 7:** F1 scores under random edge additions and deletions on five Saccharomyces cerevisiae PPI networks. Add, random insertion of edges between non-interacting protein pairs; Del, random removal of true interaction edges.

Condition	BioGRID	Collins	DIP	Krogan14k	Krogan-core
Origin	0.6706	0.8408	0.7121	0.7613	0.8045
Add 5%	0.5677	0.9004	0.7115	0.7404	0.8220
Add 10%	0.4675	0.8122	0.7254	0.6805	0.8038
Add 20%	0.3571	0.8480	0.7439	0.7192	0.8224
Del 5%	0.5737	0.9030	0.7134	0.7547	0.8469
Del 10%	0.5703	0.8974	0.6787	0.7319	0.8419
Del 20%	0.5609	0.8784	0.6313	0.7044	0.8210

**Fig 15:**
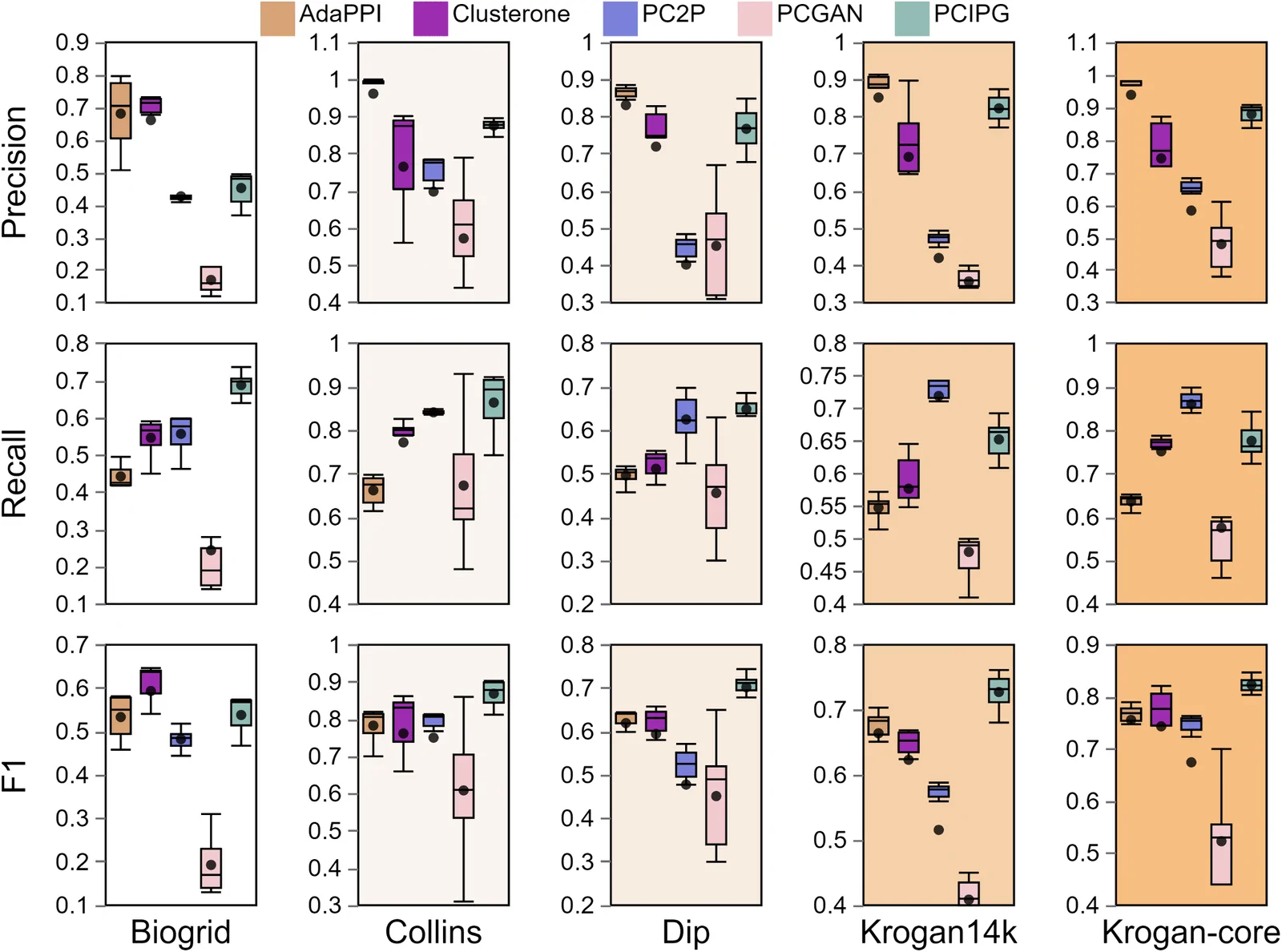
Robustness of protein complex identification methods to random edge perturbations across five Saccharomyces cerevisiae datasets. Boxplots summarize Precision, Recall and F1 for AdaPPI, ClusterONE, PC2P, PCGAN and PCIPG under random edge additions and deletions.

We compared PCIPG with four state-of-the-art protein complex identification methods. Under network perturbations (Fig 15), ClusterONE and PCGAN are particularly sensitive, whereas the remaining approaches show comparatively greater resilience. PC2P likely benefits from its non-parametric design, while AdaPPI and PCIPG reduce reliance on any single network topology by incorporating auxiliary signals, including sequence-, structure- and residue-level information.

Among these three methods, AdaPPI consistently achieves higher precision across datasets but exhibits relatively low recall, consistent with a more conservative prediction regime. In contrast, PC2P maintains high recall but lower precision, reflecting a broader “wide-net” strategy. PCIPG attains the highest recall on BioGRID, Collins and DIP, and ranks second on Krogan14k and Krogan-core. In terms of precision, it ranks second on four datasets (Collins, DIP, Krogan14k and Krogan-core). Overall, PCIPG achieves the top F1 score on Collins, DIP, Krogan14k and Krogan-core, and ranks second on BioGRID, indicating strong performance when balancing precision and recall.

The robustness of PCIPG likely stems from the complementarity of its multi-level representations and constraints. Residue physicochemistry and structural geometry provide informative priors on plausible complex boundaries, helping to mitigate local connectivity biases introduced by topological noise. Moreover, cross-level fusion buffers the model against distortions in any single evidence source, reducing performance variability. Together, the perturbation analyses suggest that PCIPG maintains a practically acceptable sensitivity to false negatives and false positives in real networks, while delivering stable, leading performance across datasets and perturbation strengths.

### 3.3 Limitations and future work

Despite these advantages, PCIPG has several limitations. First, its performance depends on the quality and coverage of input PPI networks and protein structural information. Experimentally derived interactomes remain incomplete and may contain false-positive interactions, whereas predicted structures may contain local errors, especially in flexible or disordered regions. Second, although the embedding-guided edge-completion step can mitigate missing interactions, it may also introduce false-positive edges if embedding similarity reflects shared functional context rather than direct physical association. Third, biological analyses such as GO enrichment, STRING-based network evidence, AlphaFold-based structural modeling and interface-residue prediction support the plausibility of PCIPG predictions, but they do not replace independent experimental validation. Therefore, newly predicted complexes, candidate core subunits and interface residues should be regarded as testable hypotheses. Fourth, the multi-module design of PCIPG introduces additional hyperparameters and computational cost compared with simpler topology-based methods. The biological case studies in this work should be interpreted as supportive and hypothesis-generating analyses rather than definitive experimental validation. GO/STRING enrichment, AlphaFold-based structural modeling and interface-residue prediction provide complementary evidence for the plausibility of PCIPG predictions, but they do not establish newly predicted complexes, interactions or residue-level mechanisms as experimentally confirmed. Therefore, candidate complexes, core subunits, putative interactions and predicted interface residues identified by PCIPG should be regarded as testable hypotheses. Future work will focus on stricter inductive evaluation settings, experimentally validated benchmarks, more scalable graph learning strategies and the incorporation of advanced protein language models or structure prediction confidence measures.

The computational profile of PCIPG reflects its two-level graph design. The residue-level encoder scales with the total number of residues and residue contact edges, while the PPI-level GAT scales with the number of proteins and PPI edges. In our benchmark datasets, these graph-based operations are tractable because residue graphs are processed as sparse contact graphs and the PPI networks contain at most a few thousand proteins. The main scalability bottleneck is the dense pairwise similarity calculation in the unsupervised reconstruction loss, which scales quadratically with the number of proteins. For substantially larger interactomes, this step can be replaced by mini-batch negative sampling, block-wise similarity computation or sparse candidate-pair reconstruction to reduce memory usage. These extensions would allow PCIPG to scale to larger proteome-wide networks while preserving the residue-level and PPI-level modeling strategy.

Future work may further enhance PCIPG by incorporating more advanced deep learning and graph learning strategies. For example, recent multiview neural fusion methods for protein complex identification, such as FMvPCI [37], suggest that heterogeneous biological evidence can be more flexibly integrated through neural fusion and fuzzy clustering. In addition, link-based attributed graph clustering with approximate generative Bayesian learning [38] provides a relevant direction for jointly modeling graph structure and node attributes under probabilistic assumptions. Incorporating these ideas into PCIPG may further improve its ability to integrate multi-source biological information, handle incomplete interaction networks and infer more accurate protein-complex memberships.

## 4 Methods

### 4.1 Probabilistic graph model

We modeled residue characteristics, protein features, interaction relations, and complex membership relations within a unified probabilistic graph framework (Fig 2). Assuming a protein pool comprising *N* proteins and *C* complexes, the correspondence between proteins and complexes is encoded in a distribution matrix $P\in {\mathbb{R}}^{N\times C}$ as:


$$\begin{array}{c} P_{ij}=\left\{ \begin{array}{cc} 1, & \text{if the}j\;\text{-th complex contains the}\;i\text{-th protein,}\\ 0, & \text{otherwise.} \end{array}\right. \end{array}$$ (1)

Similarly, the pairwise interaction strengths between proteins are captured by a matrix $S\in {\mathbb{R}}^{N\times N}$:


$$\begin{array}{c} S_{ij}=\left\{ \begin{array}{cc} s_{ij}, & \text{if }s_{\mathrm{ij}}\geq 0.75,\\ 0, & \text{otherwise,} \end{array}\right. \end{array}$$ (2)

where $s_{ij}$ denotes the interaction strength between protein *i* and protein *j*.

Protein-level features are encoded as $Z=(Z^{1},Z^{2},\cdots ,Z^{N}{)}^{T}$, with the feature vector of the $L_{i}$-th residue in the *n*-th protein given by:


$$\begin{array}{c} Z_{L_{i}}^{n}=(z_{l_{1}}^{n},z_{l_{2}}^{n},\cdots ,z_{l_{M}}^{n}{)}^{T}, \end{array}$$ (3)

where *M* denotes the number of physicochemical descriptors retained. The *n*-th protein representation $Z^{n}$ is obtained by aggregating $Z_{L_{i}}^{n},i=1,2,...,L_{n}$:


$$\begin{array}{c} Z^{n}=f_{1}(Z_{1}^{n},Z_{2}^{n},\cdots ,Z_{L_{n}}^{n}). \end{array}$$ (4)

We then formalized the inference procedure within a probabilistic graphical model as follows:


$$\begin{array}{c} P=f_{2}(Z,S), \end{array}$$ (5)

$$\begin{array}{c} S=f_{3}(P), \end{array}$$ (6)

where $L_{n}$ is the number of residues in the *n*-th protein, and Eq. 6 is derived under Assumption 6.1 (S1 Text).

The data flow of PCIPG proceeds from residue-level representation learning to PPI-level graph propagation and finally to protein–complex membership inference and network reconstruction. For the *n*-th protein, the feature vector of its $L_{i}$-th residue is denoted as $Z_{L_{i}}^{n}$, and the residue descriptors are organized according to the intra-chain contact map. The SAGE module implements *f*_1_ by propagating information among spatially connected residues and aggregating residue-level features into the protein-level representation $Z^{n}$. After all proteins are processed, the resulting protein representations form the matrix $Z=(Z^{1},Z^{2},\cdots ,Z^{N}{)}^{T}$. The GAT module then implements *f*_2_ by taking *Z* and the PPI strength matrix *S* as inputs, propagating information along the observed PPI network and producing the protein–complex membership matrix *P*. Each element $P_{ij}$ represents the association between protein *i* and complex *j*. Finally, the reconstruction component implements *f*_3_, in which *P* is used to reconstruct the PPI strength matrix *S* under the zero-inflated Bernoulli–Exponential likelihood. For clarity and reproducibility, the complete workflow of PCIPG is summarized in Algorithm 1. In this way, *f*_1_ converts residue-level structural and physicochemical information into protein representations, *f*_2_ integrates these representations with interaction-network context to infer protein–complex memberships, and *f*_3_ couples the inferred memberships back to the observed PPI evidence through network reconstruction.

**Algorithm 1 Overall workflow of PCIPG**

**Require:** Protein set containing *N* proteins; residue-level feature vectors $\left\{Z_{L_{i}}^{n}\right\}$; intra-chain contact maps; PPI strength matrix $S\in {\mathbb{R}}^{N\times N}$; number of complexes *C*; edge-completion threshold η; membership cutoff τ.

**Ensure:** Protein–complex membership matrix $P\in {\mathbb{R}}^{N\times C}$ and final predicted complex set 𝒞.

1:  **Residue-level representation learning**

2:  **for** each protein n=1,2,...,N **do**

3:   Organize residue-level feature vectors $\{Z_{L_{i}}^{n}{\}}_{i=1}^{L_{n}}$, defined in Eq. (3), according to the intra-chain contact map.

4:   Apply GraphSAGE propagation and nonlinear transformation to update residue representations, as described in Eq. (7)–Eq. (9).

5:   Compute residue importance scores using SAGPooling according to Eq. (10).

6:   Select informative residues through the SAGE–SAGPooling update process in Eq. (11) and Eq. (12).

7: Aggregate the retained residues by Global Mean Pooling to obtain the protein-level representation $Z^{n}$, as defined in Eq. (13).

8:  **end for**

9:  Form the protein representation matrix $Z=(Z^{1},Z^{2},...,Z^{N}{)}^{T}$, consistent with Eq. (4)

10:  **PPI-level graph propagation and membership inference**

11:  Construct the PPI adjacency matrix from the PPI strength matrix *S* according to Eq. (14).

12:  Use *Z* as the initial node-feature matrix of the PPI graph.

13:  Compute attention coefficients between interacting proteins according to Eq. (15).

14:  Propagate information along PPI edges and infer the protein–complex membership matrix

$P=f_{2}(Z,S),$

as formulated in Eq. (5).

15:  **Probabilistic network reconstruction**

16:  Reconstruct the PPI strength matrix from the membership matrix through

$S=f_{3}(P),$

as formulated in Eq. (6).

17:  Optimize PCIPG under the zero-inflated Bernoulli–Exponential reconstruction objective, as defined in Eq. (26).

18:  **Embedding-guided edge completion**

19:  Compute cosine similarity between learned protein representations in the GAT embedding space.

20:  Add candidate interactions whose similarity exceeds the threshold η.

21:  Retrain PCIPG using the completed PPI matrix when edge completion is applied.

22:  **Complex calling**

23:  Convert the final membership matrix *P* into discrete predicted complexes using cutoff τ, where protein *i* is assigned to complex *j* if $P_{ij}\geq \tau$.

24:  Remove complexes smaller than the minimum size threshold and merge highly redundant complexes.

25:  Return the final predicted complex set 𝒞.

Leveraging advances in graph neural networks (GNNs) for protein modeling [39,40], we implement *f*_1_ using GraphSAGE, thereby capturing residue-level dependencies and structural topology. The mapping *f*_2_ in Eq. 5 is framed as an overlapping community detection problem [41,42], where Graph Attention Networks (GAT) are employed to propagate information from protein features and interaction edges into complex membership assignments. Finally, for *f*_3_, we note that elements in *S* are bounded in [0,1] and exhibit marked sparsity. To address this, we adopt a zero-inflated modeling approach, applying a Bernoulli-transformed exponential distribution to capture both the discrete occurrence of interactions and the continuous variation in their strengths.

This formulation provides a unified probabilistic representation that integrates residue-level descriptors, structural embeddings, and interaction constraints, thereby enabling coherent inference of protein complexes.

To derive discrete complex predictions from the protein–complex membership matrix *P*, each column *c* was treated as a candidate complex seed, and proteins were ranked according to their membership scores $P_{i,c}$. Here, $P\in {\mathbb{R}}^{N\times C}$, where *N* denotes the number of proteins and *C* = 2000 denotes the number of candidate complex factors. Instead of using a single fixed complex size, we varied the top-*Q* selection size from *Q* = 3 to *Q* = 35 for each dataset. For each value of *Q*, the top-*Q* proteins with the highest scores in the *c*-th column of *P* were selected to form a candidate complex ${𝒞}_{c,Q}$. Candidate complexes generated over all columns and all values of *Q* were then pooled to form the protein complex set:


$$\begin{array}{c} {𝒞}_{raw}=\overset{35}{\underset{Q=3}{⋃}}\overset{2000}{\underset{c=1}{⋃}}{𝒞}_{c,Q}. \end{array}$$

This multi-*Q* extraction strategy was used to cover protein complexes with different subunit numbers, because protein complexes in curated references vary substantially in size. We did not tune a dataset-specific value of *Q*; instead, the same range Q=3,...,35 was used for all benchmarks.

### 4.2 Datasets and comparing methods

#### 4.2.1 Datasets.

Considering data availability and dataset scale, we evaluated PCIPG on eight protein–protein interaction (PPI) network benchmarks, comprising five widely used Saccharomyces cerevisiae networks (BioGRID [43], Collins [44], DIP [45], Krogan-core [46], and Krogan14k [46]) and three recently published human networks (HCT116 [47], HEK293T [47], and HuRI [48]). Following the evaluation protocol of AdaPPI, we constructed Saccharomyces cerevisiae reference complexes by integrating curated modules containing at least 3 subunits from multiple public resources, including MIPS [49], CYC2008 [50], Aloy [51], SGD [52], and TAP06 [22]. For human datasets, we used CORUM [53]—currently the most comprehensive catalog of experimentally characterized mammalian protein complexes—as the gold-standard reference set.

#### 4.2.2 Comparing methods.

Existing computational approaches for protein complex identification can be broadly categorized into unsupervised and supervised paradigms, depending on whether curated “gold-standard” complex annotations are used during model training.

Unsupervised methods do not require reference complex labels; instead, they infer putative complexes directly from PPI network organization, typically by exploiting graph topology and attribute information. As classical dense-subgraph baselines, we included MCODE [54], CMC [55], MCL [56], and ClusterONE [57]. Motivated by the core–attachment organization of protein complexes proposed by Gavin et al. [22], we additionally compared COACH [24], which explicitly models core and accessory components. To reflect recent advances in machine learning for network-based complex discovery, we further considered representative embedding/graph-learning approaches, including DPCMNE [58], Protein-AGC [59], R-GMM-VGAE [60], splp [61], PC2P [8], and TADW-SC [62]. In addition, as functional and regulatory resources have expanded with the maturation of Gene Ontology (GO) [63] and multi-omics technologies, integrative methods that incorporate external biological knowledge have gained attention; accordingly, we included GANE [64] for comparison.

Supervised methods leverage curated reference complexes as training labels and learn mappings from PPI network structure (and associated features) to protein-complex membership, enabling the prediction of novel complexes. For this category, we compared against state-of-the-art deep learning methods PCGAN [65], AdaPPI [9], and HGC [10].

### 4.3 Data preprocessing

To construct a standardized feature dataset for subsequent modeling, we developed a unified preprocessing pipeline integrating sequence retrieval, structural acquisition, residue-level feature extraction, and contact map construction. All procedures were automated via shell and Python scripts to ensure reproducibility.

First, given a protein–protein interaction (PPI) network file, we extracted the set of proteins and queried the RCSB Protein Data Bank (PDB) through the rcsbsearchapi interface. For each protein, the pipeline retrieved experimentally determined PDB files containing the protein and corresponding FASTA sequences. Redundant downloads were automatically removed, and protein identifiers were stored in a standardized text file.

Second, for proteins with available experimental structures, the pipeline parsed SOURCE and COMPND records to map genes to chains and extracted the relevant monomeric chains. Proteins without suitable experimental entries were flagged for further processing.

Third, to fill gaps in experimental coverage, the pipeline queried the UniProt ID mapping API and downloaded predicted structures from the AlphaFold Protein Structure Database. Proteins already represented in the natural structure subset were filtered out, and all remaining AlphaFold-predicted structures were parsed using the same processing procedures as above.

Fourth, residue-level physicochemical features were calculated using RDKit, including molecular weight, polar surface area (TPSA), hydrophobicity (LogP), hydrogen-bond donor/acceptor counts, aromaticity, and rotatable bonds. To address unknown residues (UNK), we constructed surrogate descriptors by averaging across all canonical amino acids, thereby ensuring numerical completeness of the feature space. Extracted features were stored in tab-delimited text files for each protein.

Finally, we constructed contact maps for both experimental and AlphaFold-predicted structures by computing inter-residue Euclidean distances at the $C_{\alpha }$ level. A threshold of 8Å was applied to define residue–residue contacts, following common practice in structural bioinformatics. These contact maps provide topological information for downstream graph-based modeling.

Collectively, this pipeline yields a consistent multi-source dataset comprising sequence files, monomeric structures, residue-level descriptors, and structural contact maps. This systematic preprocessing ensures that subsequent analyses are performed on harmonized and biologically meaningful representations of protein structure and interaction.

### 4.4 Structural feature extraction

In this step, we represent each protein as a graph in which nodes correspond to amino acid residues. Pairwise distances between residues are computed, with the relative distance between residues *i* and *j* denoted as *d*(*i*,*j*). A threshold 8Å is then applied to define the connectivity matrix *A*:


$$\begin{array}{c} A_{ij}=\left\{ \begin{array}{cc} 1, & \text{if }d(i,j)\leq 8Å\\ 0, & \text{otherwise.} \end{array}\right. \end{array}$$ (7)

Residue features are propagated through the network using the Graph Sample and AggreGatE (GraphSAGE) algorithm [66], which updates node representations by sampling and aggregating features from local neighborhoods. The update rule for the $L_{i}$-th residue node at layer *k* is:


$$\begin{array}{c} Z_{L_{i}}^{f(k)}=\sigma \left(W^{f(k)}\cdot \text{MEAN}\left(\{Z_{L_{i}}^{f(k-1)}\}\cup \{Z_{L_{j}}^{f(k-1)},\forall j\in N(i)\}\right)\right), \end{array}$$ (8)

where $W^{f(k)}$ is the *k*-th layer weight matrix, *N*(*i*) denotes the set of neighboring residues of $L_{i}$-th residue by Eq. 7, and σ(·) is a nonlinear activation.

To enhance representational capacity, we introduce a linear transformation and normalization between GraphSAGE layers, yielding the following refinement:


$$\begin{array}{c} Z_{L_{i}}^{f(k)}=Batchnorm(ReLU(W^{\left(k\right)}\cdot Z_{L_{i}}^{f(k)}+b)). \end{array}$$ (9)

This design enables the model to capture residue-level topological dependencies while maintaining stability and expressiveness during feature propagation.

### 4.5 Core locus recognition

To capture the dynamic influence of residue-level variations (e.g., mutations) on protein structures, we introduced a Self-Attention Graph Pooling (SAGPooling) module [67] built on GraphSAGE. SAGPooling identifies the most informative amino acid nodes through a top-*K* sampling strategy. Given a protein represented as $Z^{n}=(Z_{1}^{n},Z_{2}^{n},...,Z_{L_{n}}^{n}{)}^{T}$, the importance score of each residue node $Z_{L_{i}}^{n}$ is computed as:


$$\begin{array}{c} G=\sigma ({\tilde{D}}^{-\frac{1}{2}}\tilde{A}{\tilde{D}}^{-\frac{1}{2}}Z^{n}W_{att}), \end{array}$$ (10)

where $\tilde{A}=A+I$ is the augmented adjacency matrix, $\tilde{D}$ is the corresponding degree matrix, $Z^{n}\in {\mathbb{R}}^{L_{n}\times l_{M}}$ is the residue feature matrix, $W_{\text{att}}\in {\mathbb{R}}^{L_{n}\times 1}$ is a learnable scoring vector, and $G\in {\mathbb{R}}^{L_{n}\times 1}$ contains node scores. The SAGPooling ratio $r_{pool}$ denotes the proportion of residue nodes retained after pooling. In our implementation, the residue-level encoder contains three SAGPooling layers, and each layer retains 50% of the residue nodes, namely $r_{pool}=0.5$.

The residue-level update process thus integrates GraphSAGE propagation and SAGPooling selection:


$$\begin{array}{c} Z_{L_{i}}^{i_{f(k)}}=\text{SAGE}(Z_{L_{i}}^{i_{f(k-1)}}), \end{array}$$ (11)

$$\begin{array}{c} Z_{L_{i}}^{i_{f(k)}}=\text{SAGPooling}(Z_{L_{i}}^{i_{f(k)}}), \end{array}$$ (12)

where $i_{f(k)}$ is the output feature of the *k*-th layer for *i*-th protein. Finally, the remaining nodes are aggregated by Global Mean Pooling to obtain a compact protein-level representation:


$$\begin{array}{c} Z^{n}=\frac{1}{r}\sum_{i=1}^{r}Z_{L_{j_{i}}}^{n}, \end{array}$$ (13)

where $Z_{L_{j_{1}}}^{n},Z_{L_{j_{2}}}^{n},...,Z_{L_{j_{r}}}^{n}$ denote the retained residues after the last pooling step.

### 4.6 Interaction network graph learning

Building on these protein-level embeddings, we next modeled the global protein–protein interaction (PPI) network. We constructed an undirected graph where each node corresponds to a protein and edges denote experimentally verified interactions. The adjacency matrix *A* is defined as:


$$\begin{array}{c} A_{ij}=\left\{ \begin{array}{cc} 1 & \text{if }S_{ij}>0,\\ 0 & \text{otherwise.} \end{array}\right. \end{array}$$ (14)

To propagate information across the PPI graph, we applied Graph Attention Networks (GAT) [68]. Attention coefficients are computed as:


$$\begin{array}{c} \alpha_{ij}=\frac{\exp(\text{LeakyReLU}(a[WZ^{i}|WZ^{j}]))}{\sum_{n\in {𝒩}_{i}}\exp(\text{LeakyReLU}(a[WZ^{i}|WZ^{n}]))}, \end{array}$$ (15)

where *W* is a learnable transformation, *a* denotes a single-layer feedforward network, 𝒩i is the neighborhood of protein *i*, and | indicates concatenation. Node embeddings are then updated as:


$$\begin{array}{c} Z^{i}=\sigma \left(\sum_{j\in {𝒩}_{i}}\alpha_{ij}WZ^{j}\right). \end{array}$$ (16)

To improve robustness and generalization, we employed a multi-head attention mechanism, where *H* independent heads generate complementary attention weights. The final output embedding is given by:


$$\begin{array}{c} Z^{i}=\sigma \left(\frac{1}{H}\sum_{k=1}^{H}\sum_{j\in {𝒩}_{i}}\alpha_{kij}W_{k}Z^{j}\right), \end{array}$$ (17)

where $\alpha_{kij}$ and $W_{k}$ denote the attention coefficients and transformation matrix of the *k*-th head.

In summary, each GAT block computes by Eq. 17:


$$\begin{array}{c} Z^{i_{g(k)}}=\text{GAT}(Z^{i_{g(k-1)}}), \end{array}$$ (18)

followed by linear transformation and normalization to stabilize training:


$$\begin{array}{c} Z^{i_{g(k)}}=\text{BatchNorm}(\text{ReLU}(W^{g(k)}\cdot Z^{i_{g(k)}}+b)). \end{array}$$ (19)

Together, this hierarchical framework integrates residue-level pooling and network-level attention, enabling the model to capture both fine-grained structural determinants and global interaction dependencies.

### 4.7 Network enhancement learning

We introduced a network enhancement step based on similarity-driven edge completion and retraining. After the first-stage training, protein embeddings $Z^{i}$ were obtained from the GAT-based representation module, which integrates residue-level structural information and PPI-level interaction context. We then constructed a cosine similarity matrix *D*, where each element is defined as


$$\begin{array}{c} D_{ij}=\frac{Z^{i}\cdot Z^{j}}{\|Z^{i}{\|}_{2}\;\|Z^{j}{\|}_{2}}, \end{array}$$

with the numerator denoting the dot product of two protein embeddings and the denominator normalizing their magnitudes.

The similarity threshold η was determined from the distribution of $D_{ij}$ values for experimentally validated interactions in the observed PPI network. In the main setting, we used the median value, corresponding to the 0.5 quantile, as the default threshold. Candidate edges were considered only for protein pairs without an observed edge in the original PPI network. On this basis, we derived an enhanced adjacency matrix $\hat{A}$ by recovering potential false-negative edges as follows:


$$\begin{array}{c} {\hat{A}}_{ij}=\left\{ \begin{array}{cc} 1, & \text{if }A_{ij}=1\text{ or }(A_{ij}=0\text{ and }D_{ij}\geq \eta ),\\ 0, & \text{otherwise}. \end{array}\right. \end{array}$$

Thus, all originally observed PPI edges were retained, and only high-similarity unobserved pairs were added as putative missing interactions.

The model was then retrained on the enhanced network $\hat{A}$ using the same model architecture and reconstruction objective, ensuring that the learned embeddings aligned more closely with the enhanced interaction evidence. Importantly, the network enhancement step used only the observed PPI network and the learned embeddings from the first-stage model. No test complex labels or reference-complex annotations were used to determine which candidate edges were added. Finally, the protein–complex membership matrix *P* was obtained through the membership prediction module, yielding the final clustering results.

### 4.8 Training strategy

Scaling the model to large datasets introduces considerable computational challenges. Conventional training employs FP32 (single precision) arithmetic, which guarantees stable convergence but demands substantial GPU memory. On a single Quadro RTX 6000 GPU with 24 GB memory, this becomes limiting. To overcome this, we adopted a mixed-precision strategy, storing dynamic memory components—activations, gradients, and intermediate variables—in FP16 (half precision), while maintaining a master copy of model weights in FP32 for stable updates. This design reduces overall memory usage by nearly half relative to full FP32 training, enabling efficient large-scale optimization.

A key difficulty of FP16 training is its restricted dynamic range: small gradients may underflow and disrupt convergence. To address this, we incorporated loss scaling. In the scale-up phase, the loss (ΔLoss) is multiplied by 2^16^ before backpropagation, and in the scale-down phase, gradients are divided by 2^16^ and restored to FP32 before parameter updates. This stabilizes training while retaining the efficiency of FP16 arithmetic.

In practice, the initial learning rate was set to 0.001 and reduced by a decay factor γ every 100 epochs. After 2000 epochs of mixed-precision training with loss scaling, the model consistently converged, validating the robustness of this strategy.

### 4.9 Zero-inflated Bernoulli–Exponential modeling for protein–protein interactions

The motivation for using a zero-inflated Bernoulli–Exponential formulation comes from two characteristics of PPI edge-weight data. First, PPI networks are highly sparse: most protein pairs have no recorded interaction, either because they do not physically or functionally interact under the measured condition or because the interaction remains undetected. Thus, the PPI strength matrix contains an excess of zero entries. Second, for protein pairs with observed interactions, the corresponding confidence scores or interaction strengths are non-negative and typically display a right-skewed distribution. A purely binary Bernoulli model can describe whether an interaction is present, but it discards quantitative variation among non-zero edge weights. Conversely, a purely continuous model does not explicitly account for the large number of zero entries. We therefore use a Bernoulli component to model interaction occurrence and an Exponential component to model the non-zero interaction strength conditional on occurrence. This formulation allows PCIPG to distinguish whether an interaction is likely to exist from how strong the observed interaction evidence is, thereby providing a probabilistic reconstruction signal that is suitable for sparse and weighted PPI networks.

High-throughput PPI datasets typically report binarized interactions, either by directly labeling the presence or absence of an interaction or by imposing a confidence threshold below which interactions are considered unreliable [47,48]. To accommodate this property, we employed a zero-inflated model that explicitly accounts for the prevalence of non-interacting pairs. Specifically, a Bernoulli component distinguishes whether an interaction occurs, while a transformed exponential distribution describes the interaction strength when present:


$$\begin{array}{c} S\;|\;P=T\cdot g(Y), \end{array}$$ (20)

where


$$\begin{array}{c} T\sim \text{Bernoulli}(p),\;Y\sim \text{Exp}(\lambda ). \end{array}$$ (21)

The transformation function


$$\begin{array}{c} g(Y)=\frac{1}{1+Y} \end{array}$$ (22)

was found to provide a better fit to empirical distributions compared with alternative formulations. Following Assumption 6.1 (S1 Text), the Bernoulli probability *p* and exponential rate λ are parameterized by the protein–complex membership matrix *P* as:


$$\begin{array}{c} p=1-\exp(-P_{i:}P_{j:}^{T}),\;\lambda =P_{i:}P_{j:}^{T}, \end{array}$$ (23)

where $P_{i:}$ denotes the *i*-th row vector of *P*. We define the set of observed interaction edges as:


$$\begin{array}{c} E=\{(i,j)\;|\;S_{ij}>0\}. \end{array}$$ (24)

The likelihood of observing *S* given *P* can thus be expressed as:


$$\begin{array}{c} \begin{array}{cc} p(S\;|\;P) & =\prod_{\begin{array}{c} i,j=1\\ i<j \end{array}}^{N}p(S_{ij}\;|\;P)\\ & =\prod_{\begin{array}{c} i,j=1\\ i<j,(i,j)\notin E \end{array}}^{N}\exp(-P_{i:}P_{j:}^{T})\prod_{\begin{array}{c} i,j=1\\ i<j,(i,j)\in E \end{array}}^{N}[1-\exp(-P_{i:}P_{j:}^{T})]\frac{P_{i:}P_{j:}^{T}}{S_{ij}^{2}}\exp\;\left(\frac{-P_{i:}P_{j:}^{T}(1-S_{ij})}{S_{ij}}\right). \end{array} \end{array}$$ (25)

We recover the interaction matrix *S* from the protein-complex membership matrix *P* by maximizing the log-likelihood. Accordingly, the loss function is defined as:


$$\begin{array}{c} \begin{array}{cc} ℒ & =-\log p(S\;|\;P)\\ & =\sum_{\begin{array}{c} i,j=1\\ i<j,(i,j)\notin E \end{array}}^{N}P_{i:}P_{j:}^{T}-\sum_{\begin{array}{c} i,j=1\\ i<j,(i,j)\in E \end{array}}^{N}\log[1-\exp(-P_{i:}P_{j:}^{T})]-\log(P_{i:}P_{j:}^{T})+\log S_{ij}^{2}+\frac{(1-S_{ij})P_{i:}P_{j:}^{T}}{S_{ij}}. \end{array} \end{array}$$ (26)

This zero-inflated exponential formulation allows the model to simultaneously capture the sparsity of PPI networks and the graded strength of observed interactions, providing a statistically principled basis for reconstructing *S* from latent protein-complex memberships.

### 4.10 Residue importance computation

The conservation of amino acids in proteins arises from comparative analyses of homologous sequences. In multiple sequence alignments, certain residues remain invariant across divergent species or paralogous proteins over evolutionary timescales. These conserved positions are typically located at functionally essential binding sites or within structural cores, reflecting their critical roles in maintaining protein stability and activity [69]. Strong evolutionary selection pressure renders these sites more representative of the intrinsic characteristics of proteins than regions with greater variability, underscoring their importance in protein complex recognition [70].

After three GraphSAGE modules, amino acid nodes retained by the pooling layer are passed into GAT to incorporate interaction network information. During training, residues with positive self-attention scores are expected to serve as representative sites—both capturing protein-specific features for interaction learning and exhibiting a high likelihood of occupying interfacial positions. We therefore examined the ability of such key residues to function as binding sites and evolutionary conservation sites.

#### 4.10.1 Binding site prediction.

We formulate the identification of interfacial residues as an optimization problem on a bipartite residue–residue graph. Let


$$\begin{array}{c} G_{o}=(S_{o},N_{o}) \end{array}$$ (27)

denote the optimal subgraph of the bipartite graph


$$\begin{array}{c} G_{b}=(S_{b},N_{b}), \end{array}$$ (28)

where vertices correspond to residues and edge weights represent intra-chain distances. The objective is defined as:


$$\begin{array}{c} \min\sum_{s\in S_{o}}c_{s}+\alpha \sum_{n\notin N_{o}}w_{n}+\beta \cdot h, \end{array}$$ (29)

where $c_{s}$ is the distance-based edge weight, $w_{n}$ is the residue-level attention score assigned by Eq. 12, and h is the number of disconnected components. Intuitively, this formulation selects spatially proximal residues with high importance scores as interaction interfaces, while minimizing the number of disconnected interface regions. As this problem is NP-hard [71], we adopted a Steiner Forest graph optimization strategy, implemented using the PCSF R package [72], which introduces an auxiliary vertex to ensure graph connectivity.

Because PCSF was originally designed for biological networks with edge weights in the range [0,1] [73,74], amino acid distances were normalized using a max–min transformation:


$$\begin{array}{c} c_{s}=\frac{d_{s}-d_{\min}}{d_{\max}-d_{\min}}, \end{array}$$ (30)

where $d_{s}$ denotes the distance between two residues connected by edge *s*. Parameters were set to α=1 and β=8 in Eq. 29.

#### 4.10.2 Conservation site prediction.

To evaluate evolutionary conservation, we generated all protein pairs in the Collins dataset and queried the RCSB PDB for complexes containing each pair. For proteins present in complexes, amino acid sequences of target chains were extracted and homologous sequences were retrieved using BLASTP searches [75] against the NCBI non-redundant (nr) database. Homologs were aligned with Clustal Omega [76], and residue-level conservation scores (CS) were computed as:


$$\begin{array}{c} CS=1-\frac{N_{\text{unique}}}{N_{\text{total}}}, \end{array}$$ (31)

where *N*_unique_ denotes the number of distinct amino acids at a given alignment column and *N*_total_ is the total number of aligned sequences.

To assess statistical significance, a paired *t*-test with unequal variance was applied at the protein level:


$$\begin{array}{c} H_{0}:\mu_{0}=\mu_{1},\;H_{1}:\mu_{0}>\mu_{1}, \end{array}$$ (32)

where $\mu_{0}$ and $\mu_{1}$ represent the mean conservation scores of key and non-key residues, respectively. Only proteins with at least two residues in each category were considered. Finally, conservation scores across all proteins were pooled, and a global one-tailed two-sample *t*-test (*p* < 0.05) was conducted to determine whether key residues exhibit significantly higher conservation than non-key residues.

### 4.11 Module and core component analysis in protein complexes

#### 4.11.1 Module analysis.

As a downstream analysis of the protein-complex membership matrix *P*, module detection seeks to group proteins that share similar community assignments. Proteins assigned to the same module are expected to share similar binary labels indicating co-membership within a complex, and thus exhibit comparable values in the protein-complex membership matrix. Accordingly, we used the protein-complex membership matrix *P*, derived from GAT-based protein embeddings, as the sample–feature matrix for hierarchical clustering of proteins into modules.

For performance evaluation, in addition to standard complex identification metrics, we employed normalized mutual information (NMI), one of the most widely used external measures of clustering quality, defined in Eq. 44.

#### 4.11.2 Core component analysis.

As another downstream application of the protein-complex membership matrix *P*, we performed core component analysis to identify proteins that stabilize each module. To this end, we first grouped predicted complexes into clusters based on subunit overlap, thereby capturing sets of highly similar complexes that mimic “isoforms”. Within each cluster, proteins with high recurrence frequencies were designated as candidate core components.

The degree of a protein was defined as the number of complexes in which it appeared, whereas the degree of a complex was defined as the sum of the degrees of all its constituent proteins. We initially ranked all predicted complexes by degree and selected the top *o* as the preliminary core set. Thereafter, for each complex in the core set, we merged those complexes whose overlap score with it exceeded the threshold 0.25, as described in [4]. The overlap score was calculated as:


$$\begin{array}{c} Overlap(A,B)=\frac{|A\cap B{|}^{2}}{\left|A|\times |B\right|}, \end{array}$$ (33)

where *A* and *B* denote two protein complexes.

Finally, to pinpoint core proteins within each cluster, we performed statistical enrichment analysis. Specifically, for each protein, a *t*-test compared its frequency of occurrence within a given cluster against its frequency across all other clusters. The null hypothesis assumed equal mean occurrence, whereas the alternative hypothesis posited significant enrichment within the focal cluster. Proteins exhibiting significantly higher recurrence in a given cluster were designated as the core components of that cluster.

### 4.12 Protein mutation identification

To further assess the capacity of PCIPG in modeling the impact of single-protein mutations on interaction networks, we applied it to the identification of mutation-specific interaction landscapes. The dataset was obtained from reference [22], which experimentally determined the protein–protein interaction (PPI) networks of the ΔF508 CFTR mutant and the wild-type (WT) CFTR. The mutant and WT networks comprise 951 and 759 interactions, respectively, with 511 shared edges between them. These interactions encompass both direct and indirect partners of CFTR.

Using the 511 overlapping interactions as a common reference, we trained the model independently on the WT and mutant CFTR networks and performed interaction inference with quantile thresholds η. The Jaccard similarity coefficient defined as:


$$\begin{array}{c} \text{Jaccard}=\frac{\left|Pred\cap Truth\right|}{\left|Pred\cup Truth\right|}. \end{array}$$ (34)

More specifically, we jointly account for both WT and mutant networks:


$$\begin{array}{c} \Delta_{\text{Jaccard}}=\frac{\left|D_{ph}\cap D_{h}\right|}{\left|D_{ph}\cup D_{h}\right|}+\frac{\left|D_{pd}\cap D_{d}\right|}{\left|D_{pd}\cup D_{d}\right|}-\frac{\left|D_{ph}\cap D_{d}\right|}{\left|D_{ph}\cup D_{d}\right|}-\frac{\left|D_{pd}\cap D_{h}\right|}{\left|D_{pd}\cup D_{h}\right|}, \end{array}$$ (35)

where $D_{ph}$ and $D_{pd}$ denote the interaction networks predicted by models trained on the WT and mutant structures, respectively, while $D_{h}$ and $D_{d}$ correspond to the experimentally observed WT and mutant networks.

This procedure enabled systematic evaluation of how well the model captured mutation-induced rewiring of protein interaction landscapes, providing a principled basis for identifying mutation-specific perturbations in the PPI network.

### 4.13 GO semantic similarity calculation

To evaluate the functional coherence of predicted protein complexes, we collected Gene Ontology (GO) annotations for the corresponding gene sets and assessed their semantic similarity across the three GO domains: Biological Process (BP), Molecular Function (MF), and Cellular Component (CC). Genes with higher semantic similarity are more likely to participate in related biological processes, and their encoded proteins are correspondingly more likely to assemble into functional complexes.

Semantic similarity scores between all gene pairs were computed using the GOSemSim package [77]. For a set of $N_{G}$ genes, we constructed a score matrix *SC*, where each entry $SC_{ij}$ represents the semantic similarity score between gene *i* and gene *j*. The overall semantic similarity of a gene set was quantified by the normalized Frobenius norm:


$$\begin{array}{c} Score_{GO}=\frac{||SC|{|}_{F}}{N_{G}}=\frac{\sqrt{\sum_{i,j=1}^{N_{G}}SC_{ij}^{2}}}{N_{G}}, \end{array}$$ (36)

where $||SC|{|}_{F}$ denotes the Frobenius norm of the score matrix.

To provide a reference baseline, random complex datasets were generated following the experimental protocols of Qi et al. [18] and Wang et al. [19]. Specifically, nodes were randomly sampled from the PPI network to construct synthetic complexes, while enforcing that their size distribution followed the power-law pattern observed in curated gold-standard complexes. The ratio of random to gold-standard complexes was fixed at 5:1 to ensure sufficient contrast.

In addition, to provide a more stringent control, we generated size- and degree-matched random protein sets. For each evaluated complex *C*, we generated a random set *R* with |*R*| = |*C*|. Let *d*(*v*) denote the degree of protein *v* in the corresponding PPI network. Proteins were grouped into degree bins according to their PPI degrees. For each protein v∈C, a replacement protein *u* was randomly sampled from the same degree bin as *v*. If no candidate protein was available in the same bin, sampling was performed from the nearest non-empty degree bin. Duplicate proteins within the same random set were not allowed. This procedure preserves the complex-size distribution and approximates the degree distribution of the original complexes. GO semantic similarity scores were then computed for these matched random sets using the same GOSemSim-based procedure as for curated and PCIPG-predicted complexes.

### 4.14 Computational complexity analysis

We further analyzed the computational complexity of PCIPG. Let *N* denote the number of proteins, *C* the number of complexes, $L_{n}$ the number of residues in the *n*-th protein, *M* the number of residue descriptors, *d* the hidden dimension, *E* the number of non-zero entries in the PPI strength matrix *S*, and $E_{n}^{res}$ the number of residue-contact edges in the intra-chain contact map of the *n*-th protein. In the residue-level representation learning step, the SAGE module implements *f*_1_ by propagating information on residue-contact graphs. Its time complexity is approximately


$$\begin{array}{c} O\left(\sum_{n=1}^{N}E_{n}^{res}d\right), \end{array}$$

where the dominant cost comes from message passing among spatially connected residues. The initial transformation of residue descriptors contributes


$$\begin{array}{c} O\left(\sum_{n=1}^{N}L_{n}Md\right). \end{array}$$

After residue-level aggregation, the protein representations form $Z=(Z^{1},Z^{2},...,Z^{N}{)}^{T}$.

In the PPI-level graph propagation step, the GAT module implements *f*_2_ using *Z* and the PPI strength matrix *S*. Since message passing is performed along the non-zero PPI entries, the computational cost is approximately


$$\begin{array}{c} O(Ed), \end{array}$$

or *O*(*E H d*) when *H* attention heads are used. The MLP decoder that maps protein representations to the protein–complex membership matrix $P\in {\mathbb{R}}^{N\times C}$ requires


$$\begin{array}{c} O(NCd) \end{array}$$

operations.

The reconstruction component implements *f*_3_, which reconstructs the PPI strength matrix *S* from the membership matrix *P* under the zero-inflated Bernoulli–Exponential likelihood. If the reconstruction loss is evaluated on observed PPI entries and sampled unobserved pairs, its complexity is


$$\begin{array}{c} O((E+E^{-})C), \end{array}$$

where *E*^-^ denotes the number of sampled unobserved protein pairs. If all protein pairs are used, the corresponding cost becomes *O*(*N*^2*C*^).

For embedding-guided edge completion, exhaustive cosine-similarity calculation between all protein representations requires


$$\begin{array}{c} O(N^{2}d). \end{array}$$

This term is only required when edge completion is applied and can be reduced by restricting candidate pairs or using approximate nearest-neighbor search. Finally, converting the membership matrix *P* into discrete predicted complexes requires


$$\begin{array}{c} O(NC). \end{array}$$

Therefore, when sampled unobserved pairs are used for reconstruction, the overall time complexity of PCIPG can be summarized as


$$\begin{array}{c} O\left(\sum_{n=1}^{N}L_{n}Md+\sum_{n=1}^{N}E_{n}^{res}d+EHd+NCd+(E+E^{-})C+N^{2}d\right), \end{array}$$

where the *N*^2^*d* term corresponds to the optional exhaustive edge-completion step. Without edge completion, this term is omitted. The main memory cost arises from storing residue-level representations, residue-contact maps, the PPI strength matrix *S*, protein representations *Z*, and the membership matrix *P*, giving an approximate space complexity of


$$\begin{array}{c} O\left(\sum_{n=1}^{N}L_{n}d+\sum_{n=1}^{N}E_{n}^{res}+E+Nd+NC\right). \end{array}$$

### 4.15 Hyperparameter settings and tuning protocol

To improve reproducibility, we summarize the key hyperparameters and their tested ranges in Table 8. PCIPG was trained without curated protein-complex labels in its reconstruction objective; the loss function depended only on residue-level features, protein structural information, and the observed PPI network. However, no independent labeled validation set was used for model selection. BioGRID was designated as a single development dataset for performance-informed architecture and residue-feature selection. The numbers of SAGE and GAT modules, hidden dimension, learning rate, dropout rate, SAGPooling ratio, and residue-feature composition were examined on BioGRID using protein-complex identification metrics, training stability, computational efficiency, and biological interpretability. Thus, although BioGRID complex annotations were not used for gradient-based optimization, their evaluation metrics were consulted when selecting among candidate configurations.

**Table 8:** Key hyperparameters and design choices of PCIPG.

Hyperparameter	Default value	Tested range	Selection rationale
Number of SAGE modules	3	{1,2,3,4}	Selected based on model performance.
Number of GAT modules	3	{1,2,3,4}	Selected based on model performance for PPI-level graph propagation.
Hidden dimension	512	{256,512,768}	Chosen to balance representation capacity and computational cost.
Learning rate	0.001	{0.1,0.01,0.001}	Selected according to model performance.
Dropout rate	0.5	{0.2,0.5,0.7}	Used for regularization and selected based on model performance.
SAGPooling ratio	0.5	{0.2,0.5,0.7}	Selected to retain informative residues while reducing noisy residue-level features.
Contact-map threshold	8 Å	–	Chosen based on common structural-contact definitions.
Residue descriptor groups	Six groups retained	Seven groups tested	Selected based on residue-feature ablation; the electronic-property group was removed due to redundancy.
High-confidence edge threshold η	0.95	{0.90,0.93,0.95,0.97}	Selected based on edge-completion sensitivity analysis to avoid excessive graph densification.
Membership cutoff *Q*	3-35	–	Used to convert the membership matrix *P* into discrete complexes.
Overlap threshold *m*	0.3	–	Used for redundancy pruning and module/core grouping; selected based on benchmark conventions.

After the development-stage configuration was finalized, the same architecture, residue representation, and training settings were fixed and applied to Collins, DIP, Krogan-core, Krogan14k, HCT116, HEK293T, and HuRI without further architecture- or feature-level tuning against their reference complexes. Consequently, BioGRID results are reported as development-dataset performance, whereas the remaining datasets provide cross-dataset evaluation of the fixed configuration.

Post-processing parameters were not optimized independently for each benchmark. For complex generation, the same predefined range Q=3,...,35 was applied to every dataset, and candidate complexes generated across all values of *Q* were pooled. The normalized-association threshold used for evaluation and the overlap criterion used for redundancy removal followed established protein-complex benchmark conventions. The edge-completion threshold η was evaluated through sensitivity analyses on the three human datasets themselves; accordingly, edge-completed results are presented as secondary within-dataset analyses, whereas results from the original PPI networks constitute the primary human benchmark comparisons.

### 4.16 Evaluation indices for protein complex identification

To comprehensively assess the accuracy of protein complex identification, we adopted the evaluation framework used in AdaPPI [9], employing *Precision*, *Recall*, *ACC*, and *F*1 as primary metrics. For each predicted complex and reference complex, we computed the normalized association score to quantify their overlap. A predicted complex was considered to match a reference complex when the normalized association exceeded the predefined threshold. Precision, Recall and F1-score were calculated based on the matched predicted and reference complexes, while ACC was computed from PPV and sensitivity following the standard evaluation protocol for protein complex identification. Their definitions are given in Eqs. 37–43.

The positive predictive value (PPV) is defined as:


$$\begin{array}{c} PPV=\frac{\sum_{j=1}^{\left|P_{c}\right|}\max_{i=1}^{\left|B_{c}\right|}T_{ij}}{\sum_{j=1}^{\left|P_{c}\right|}N_{j}}, \end{array}$$ (37)

where $P_{c}$ and $B_{c}$ denote the sets of predicted and reference complexes, respectively; |·| represents set cardinality; $N_{j}$ is the number of proteins in predicted complex *j*; and $T_{ij}$ is the number of shared proteins between predicted complex *j* and reference complex *i*.

The accuracy index *ACC* is derived from PPV and sensitivity (*Sn*) as:


$$\begin{array}{c} ACC=\sqrt{Sn\times PPV}, \end{array}$$ (38)

where sensitivity is defined as:


$$\begin{array}{c} Sn=\frac{\sum_{i=1}^{\left|B_{c}\right|}\max_{j=1}^{\left|P_{c}\right|}T_{ij}}{\sum_{i=1}^{\left|B_{c}\right|}M_{i}}, \end{array}$$ (39)

with $M_{i}$ denoting the number of proteins in reference complex *i*.

To further evaluate prediction quality, we employed pairwise matching criteria based on normalized association (NA). Specifically:


$$\begin{array}{c} Precision=\frac{\left|\{p\in P_{c}|\exists b\in B_{c},NA(p,b)\geq \rho \}\right|}{\left|P_{c}\right|}, \end{array}$$ (40)

$$\begin{array}{c} Recall=\frac{\left|\{b\in B_{c}|\exists p\in P_{c},NA(p,b)\geq \rho \}\right|}{\left|B_{c}\right|}, \end{array}$$ (41)

$$\begin{array}{c} F1=\frac{2\times Precision\times Recall}{Precision+Recall}, \end{array}$$ (42)

where ρ is set to 0.25 following Hu et al. [4]. The normalized association is given by:


$$\begin{array}{c} NA(p,b)=\frac{|V_{p}\cap V_{b}{|}^{2}}{\left|V_{p}|\times |V_{b}\right|}, \end{array}$$ (43)

with $V_{p}$ and $V_{b}$ representing the sets of subunits in complexes *p* and *b*, respectively.

In addition, we used Normalized Mutual Information (NMI) to assess clustering quality in module identification. Let $𝒯=\{{𝒯}_{1},{𝒯}_{2},...,{𝒯}_{t}\}$ be the ground-truth partition and $𝒞=\{{𝒞}_{1},{𝒞}_{2},...,{𝒞}_{p}\}$ be the clustered partition, with *t* and *p* denoting the numbers of true and predicted clusters, respectively. NMI is defined as:


$$\begin{array}{c} NMI(𝒞,𝒯)=\frac{2\sum_{ij}n_{ij}\log\frac{n\;n_{ij}}{a_{i}b_{j}}}{-\sum_{i}a_{i}\log\frac{a_{i}}{n}-\sum_{j}b_{j}\log\frac{b_{j}}{n}}, \end{array}$$ (44)

where $n_{ij}=|{𝒯}_{i}\cap {𝒞}_{j}|$, $a_{i}=|{𝒯}_{i}|$, $b_{j}=|{𝒞}_{j}|$, and *n* is the total number of proteins. NMI values range from 0 to 1, with 1 indicating perfect agreement between predicted and reference partitions.

### 4.17 Filtering high-confidence predicted complexes

To ensure the reliability of predicted protein complexes, we introduced the internal connectivity ratio as a quantitative quality metric. This index measures the cohesiveness of a protein set within the protein–protein interaction (PPI) network. For a given protein set *P*, the internal connection count is defined as the number of edges in the PPI network whose endpoints both lie in *P*. The internal connectivity ratio *R* is then calculated as the proportion of observed internal connections relative to the theoretical maximum for the set.

Formally, for a set *P* with $m_{p}$ proteins, the maximum number of possible internal edges is (mp2). If the actual number of internal edges is denoted as *L*, the internal connectivity ratio *R* is defined as:


R=L(mp2). (45)

This metric provides a direct indicator of structural compactness, with higher values reflecting stronger internal associations within the predicted complex. To minimize false positives, we applied adaptive post-filtering criteria according to complex size. For trimers and tetramers, only those with R≥1 were retained, thereby enforcing strict internal cohesion for small assemblies. For complexes with five or more subunits, a relaxed threshold of R≥0.2 was adopted, acknowledging that larger assemblies may exhibit lower absolute densities of connections while still maintaining biological plausibility.

For each benchmark dataset, we recorded the number of candidate complexes extracted from the learned protein–complex membership matrix before internal-network filtering, denoted by $N_{extract}$. Each extracted candidate was then evaluated using the internal connectivity ratio defined in Eq. 45. Candidates that did not satisfy the size-dependent internal-connectivity criterion were removed, and all candidates that passed the criterion were retained as the final predicted complex set used for benchmark evaluation. We denote the number of retained predictions by $N_{retain}$. The number removed during this post-processing stage is therefore $N_{extract}-N_{retain}$. No gold-standard annotation was used to determine which candidates were retained. Detailed information can be found in Table 9.

**Table 9:** Stage-wise statistics for converting the protein–complex membership matrix into discrete predicted complexes.

Dataset	Raw Complexes	Filtered Complexes	Matched Gold-standard complexes
BioGRID	49694	36577	775
Collins	52559	30638	311
DIP	60710	14000	586
Krogan-core	59411	19033	325
Krogan14k	59162	23460	381
HCT116	58805	6327	355
HEK293T	57986	14341	1477
HuRI	19193	324	260

Comparable pre-filtering candidate counts could not be reconstructed for every baseline method because several implementations provide only their final predicted complex sets. We therefore do not claim a direct stage-wise comparison of candidate-pool sizes across all methods. However, all retained PCIPG complexes are included in the calculation of Precision, Recall, F1 and ACC, and the reported value of $N_{retain}$ provides the complete size of the PCIPG prediction set used for evaluation.

## 5 Conclusion

In this study, we developed PCIPG to integrate structural and biochemical information with network context for protein complex identification within a unified probabilistic framework. By explicitly linking residue-level representations to protein embeddings and complex membership, and by reconstructing interaction strengths through a zero-inflated Bernoulli–Exponential model, PCIPG offers a principled way to couple complex co-membership with the PPI network.

Methodologically, PCIPG leverages intra-chain contact-map message passing and pooling to highlight interaction-relevant residues, while graph attention over the PPI network integrates global interaction context and yields a probabilistic protein-complex membership matrix for protein complex identification.

Experiments showed PCIPG delivers strong performance on widely used Saccharomyces cerevisiae benchmarks, and its interpretability is supported by the enrichment of high-scoring residues at binding interfaces and by case evidence that the inferred interaction landscape responds to perturbations such as CFTR mutation.

In human interactomes—where incompleteness is more severe—embedding-driven edge completion based on cosine similarity increases network connectivity and consistently improves complex identification results. Functional evaluation further indicates that complexes predicted after edge completion exhibit higher GO semantic coherence.

Overall, PCIPG advances protein complex identification by bridging residue-scale signals and interactome-scale structure, while providing an interpretable route to prioritize interface residues and refine predictions under incomplete interaction data.

## Supporting information

S1 Table Dataset statistics and edge-completion sensitivity analysis. This table summarizes the basic statistics of the protein–protein interaction datasets used in this study, including the number of proteins, interactions, reference complexes, and the PPI-to-complex ratio before and after embedding-guided network enhancement. The PPI-to-complex ratio provides a dataset-level summary of the amount of interaction evidence available per reference complex under the original and enhanced network settings.
(XLSX)

S2 Table Full performance comparison on human protein–protein interaction datasets. This table reports the complete performance comparison of PCIPG and competing protein complex identification methods on three human datasets, HCT116, HEK293T, and HuRI. Evaluation metrics include precision, recall, F1-score, and accuracy.
(XLSX)

S3 Table Reference core and module annotations used for core–module analysis. This table provides the gold-standard core and module annotations used to evaluate PCIPG in the core–module analysis. It includes curated core complexes, module annotations, protein members, complex sizes, and corresponding PCIPG-predicted candidates where available.
(XLSX)

S4 Table CFTR wild-type, mutant, and temperature-dependent interaction data. This table contains the interaction data used for the CFTR case study, including wild-type CFTR interactions, disease-state interactions, temperature-dependent interaction profiles under the disease condition, and shared wild-type–disease genes.
(XLSX)

S5 Table Collins dataset node-weight scores and protein index mapping. This table provides the node-weight scores used in the Collins dataset analysis, together with the mapping between protein identifiers and internal node indices. These data support the residue- and protein-level analyses performed on the Collins benchmark.
(XLSX)

S6 Table Relationship between shared complex membership and PPI occurrence across datasets. This table reports the association between the number of shared predicted complex memberships and the occurrence of protein–protein interactions across yeast and human datasets. For each dataset, it provides the number of protein pairs with a given shared-membership count, the number of corresponding PPI pairs, and the resulting PPI ratio.
(XLSX)

S1 TextThis file provides supplementary analyses supporting the methodological clarification, robustness evaluation, computational assessment, and residue-level validation of PCIPG.It first describes the training, validation, and evaluation protocol, clarifying that PCIPG is trained as an unsupervised protein complex identification framework and that curated protein-complex annotations are used only for final evaluation rather than model training. It then reports parameter sensitivity analyses for key hyperparameters, including the number of SAGE and GAT layers, hidden dimension, learning rate, SAGPooling ratio, and dropout rate. In addition, the file presents stability analyses based on 20 independent runs with different random seeds, including mean ± standard deviation and 95% confidence intervals. Runtime and GPU memory usage across benchmark datasets are also summarized to evaluate computational scalability. Finally, the supplementary file provides additional residue-level evaluation by reporting interface-residue prediction recall across all compared methods. Furthermore, the supplementary file adds an empirical analysis supporting the protein co-membership assumption by quantifying the relationship between repeated co-occurrence of protein pairs in curated complexes and their interaction support across yeast and human PPI datasets. It also documents the updated source-code repository and README file, including the overall workflow, model architecture, loss functions, training and inference procedures, data formats, tensor shapes, supported datasets, and example command-line usage to improve reproducibility.(PDF)
