# Supplementary material for: PCIPG: A comprehensive framework for protein complex identification based on a probabilistic graphical model
Source: PLoS Comput Biol. 2026 Sep 3;22(9):e1014698. doi: 10.1371/journal.pcbi.1014698 (PMC13577533; doi:10.1371/journal.pcbi.1014698)
Supplement: Supplementary file 1 [file pcbi.1014698.s007.pdf]

Supplementary Materials

1 Training, validation and evaluation protocol

PCIPG is trained as an unsupervised protein complex identification framework rather than a supervised or semi-supervised complex classifier. During training, the model uses residue-level physicochemical descriptors, intra-chain residue contact maps and the available PPI network. The PPI network is used as the message-passing topology for protein-level graph learning and as the reconstruction target for the unsupervised objective. Curated protein-complex annotations are not used as training labels.

Therefore, the main PCIPG training procedure does not involve a conventional train/validation/test split over labeled protein complexes. For each benchmark dataset, we trained PCIPG on the available PPI network and protein structural features, inferred the protein–complex membership matrix, and then converted this matrix into discrete predicted complexes. The predicted complexes were subsequently evaluated against curated gold-standard complexes using precision, recall, F-score and Acc. Gold-standard complexes were used only for evaluation and were not used to optimize model parameters.

For hyperparameter analysis, ablation experiments and sensitivity tests, we applied the same evaluation protocol consistently across compared settings. These analyses were used to examine the influence of model components and parameter choices, but the gold-standard complex labels were not used as supervised training targets. For embedding-guided network enhancement, putative edges were added based only on the learned protein embeddings and the available PPI network. No gold-standard complex annotations were used in the edge-completion step. This protocol avoids label leakage from curated complex annotations into model training.

2 Parameter sensitivity analysis

We evaluated the sensitivity of PCIPG to key hyperparameters by varying the number of SAGE and GAT layers, hidden dimension, learning rate, SAGPooling ratio, and dropout rate. For each setting, we report precision, recall, F1 score, and accuracy on the benchmark dataset.

Table A. Parameter sensitivity analysis for the number of SAGE layers.

Layers	Precision	Recall	F1	Accuracy
1	0.8120	0.7167	0.7614	0.5430
2	0.7916	0.7360	0.7628	0.5531
3	0.7799	0.8939	0.8330	0.6461
4	0.7708	0.8325	0.8005	0.6124

3 Stability analysis

To assess the stability of PCIPG, we performed 20 independent runs with different random seeds for each benchmark dataset while keeping all model and post-processing

Table B. Parameter sensitivity analysis for the number of GAT layers.

Layers	Precision	Recall	F1	Accuracy
1	0.7090	0.5945	0.6467	0.5432
2	0.7617	0.8164	0.7881	0.5704
3	0.7799	0.8939	0.8330	0.6461
4	0.7219	0.8196	0.7677	0.5608

Table C. Parameter sensitivity analysis for the hidden dimension.

Dimension	Precision	Recall	F1	Accuracy
256	0.7839	0.8100	0.7967	0.6828
512	0.7799	0.8939	0.8330	0.6461
768	0.7836	0.8582	0.8192	0.6815

Table D. Parameter sensitivity analysis for the learning rate.

Learning rate	Precision	Recall	F1	Accuracy
0.1	0.7548	0.6077	0.6733	0.5082
0.01	0.5095	0.6174	0.5583	0.4386
0.001	0.7799	0.8939	0.8330	0.6461
0.0001	0.8162	0.6881	0.7467	0.4926

Table E. Parameter sensitivity analysis for the SAGPooling ratio.

Pooling ratio	Precision	Recall	F1	Accuracy
0.2	0.9482	0.6206	0.7502	0.5172
0.5	0.7799	0.8939	0.8330	0.6461
0.7	0.8474	0.7717	0.8078	0.6314

Table F. Parameter sensitivity analysis for the dropout rate.

Dropout rate	Precision	Recall	F1	Accuracy
0.2	0.7481	0.8260	0.7851	0.5540
0.5	0.7799	0.8939	0.8330	0.6461
0.7	0.8422	0.7457	0.7910	0.5466

settings fixed. The baseline methods were not rerun under the same 20-seed protocol; therefore, the repeated-run analysis is used solely to characterize the variability of PCIPG and not to conduct seed-matched statistical tests against the baselines.

Table G reports the mean ± standard deviation of precision, recall, F-score and Acc. Table H further reports the 95% confidence interval of the F-score. These results indicate that PCIPG achieves stable performance across random seeds, with relatively small run-to-run variation.

Table G. Repeated-run performance of PCIPG across benchmark datasets in 20 independent runs. Results are reported as mean ± standard deviation.

Dataset	Precision	Recall	F-score	Acc
BioGRID	0.5898 ± 0.0346	0.8195 ± 0.0382	0.6801 ± 0.0369	0.5862 ± 0.0327
DIP	0.6669 ± 0.0475	0.7523 ± 0.0418	0.6942 ± 0.0354	0.5890 ± 0.0376
Krogan14k	0.7452 ± 0.0432	0.6947 ± 0.0305	0.7220 ± 0.0448	0.6035 ± 0.0297
Krogan-core	0.8446 ± 0.0398	0.8157 ± 0.0463	0.8098 ± 0.0389	0.6591 ± 0.0335
Collins	0.7661 ± 0.0316	0.9072 ± 0.0407	0.8375 ± 0.0469	0.6498 ± 0.0358
HCT116	0.8298 ± 0.0284	0.5124 ± 0.0441	0.6320 ± 0.0485	0.4559 ± 0.0362
HEK293T	0.7662 ± 0.0412	0.4274 ± 0.0349	0.5554 ± 0.0395	0.5122 ± 0.0478
HuRI	0.6716 ± 0.0457	0.3104 ± 0.0374	0.4279 ± 0.0491	0.3923 ± 0.0338

Table H. The 95% confidence interval of repeated-run F-score performance.

Dataset	95% CI of F-score
BioGRID	$[0.6628, 0.6974]$
DIP	$[0.6776, 0.7108]$
Krogan14k	$[0.7010, 0.7430]$
Krogan-core	$[0.7916, 0.8280]$
Collins	$[0.8155, 0.8595]$
HCT116	$[0.6093, 0.6547]$
HEK293T	$[0.5369, 0.5739]$
HuRI	$[0.4049, 0.4509]$

4 Runtime and memory usage

Table I. Runtime and GPU memory usage of PCIPG on the evaluated datasets. Runtime denotes the wall-clock training time under the reported experimental setting. Peak memory denotes the maximum GPU memory allocated during training. N is the number of proteins, T is the total number of residues, E_{ppi} is the number of PPI edges, and E_{res} is the total number of residue-level contact edges.

Dataset	N	T	E_{ppi}	E_{res}	Runtime	Peak GPU memory
BioGRID	2077	895057	24721	12521144	1223.81s	4.601GB
Collins	902	397592	4351	6654838	854.49s	2.330GB
DIP	1604	713135	5805	10599294	1270.76s	3.782GB
Krogan14k	1025	478405	4230	7462678	878.21s	2.639GB
Krogan-core	881	395570	2723	6669496	844.96s	2.331GB
HCT116	688	201615	9106	11117453	1273.52s	3.249GB
HEK293T	2201	855313	18809	35024914	1663.53s	10.301GB
HuRI	529	151732	9303	9781692	609.81s	2.838GB

5 Additional residue-level evaluation

To provide a more complete evaluation of residue-level prediction performance, we further report residue-level recall score in addition to precision (Tab. J). Since precision depends on the number of residues predicted as positive, all compared methods were evaluated under the same predicted residue numbers. Recall measures the fraction of annotated interface residues that are successfully recovered by each method. Therefore, the newly added recall score tables provide complementary information to the precision results and allow a more balanced assessment of residue-level prediction performance.

Table J. Comparison of interface-residue prediction recall (%) for PCIPG and baseline methods across representative protein complexes.

PDB ID	Protein (Gene)	PCIPG	DeepPPISP	PGT	HN-PPISP
8EKI	YDR498C	**31.58**	24.63	28.14	19.52
	YGL098W	**22.22**	18.73	16.42	20.08
6YGD	YPR051W	27.59	21.36	**44.82**	39.24
	YEL053C	**24.32**	12.47	8.93	15.31
6VZG	YPR034W	0.00	7.84	**12.63**	5.91
	YGR275W	**87.50**	74.18	42.57	39.36
9GD4	YLR314C	6.90	11.26	**73.41**	14.68
	YHR107C	**18.87**	13.24	14.57	12.86
5L52	YGL011C	**23.08**	17.35	20.16	12.44
	YML092C	**23.08**	19.42	15.73	11.58
5L52	YGR135W	**50.00**	43.61	31.28	45.24
	YER012W	**85.71**	35.49	76.32	47.83
5L52	YGR135W	7.14	**72.56**	5.38	13.72
	YML092C	**33.33**	28.46	25.94	30.17
5L52	YGR135W	22.22	10.81	41.67	**46.58**
	YER094C	**83.33**	41.52	19.64	74.86

6 Empirical support for the protein co-membership assumption

Assumption 6.1. *There is a positive correlation between the strength of interaction and the number of complexes that two proteins participate in. That is, if two proteins participate in more complexes, the greater the probability that the two proteins have an interaction edge and tend to have a higher interaction strength.*

To empirically examine Assumption 6.1, we quantified how often two proteins co-occur across curated complexes and asked whether this recurrence is supported by independent PPI evidence. Specifically, for each interactome, we enumerated all protein pairs that appear together in at least one gold-standard complex and grouped them by their exact co-occurrence count X, defined as the number of distinct complexes in which the same pair is jointly observed. For each X, we then computed the fraction of these co-occurring pairs that are present as an interaction edge in the corresponding PPI network (S6 Table). As shown in Fig. A, the interaction-supported fraction increases with X across both yeast and human datasets, indicating that repeated complex co-membership enriches for protein pairs that are more likely to be connected in the measured interactome. This monotonic trend provides quantitative support for Assumption 6.1, suggesting that complex recurrence can serve as a proxy signal for interaction reliability and, by extension, interaction strength when the underlying PPI

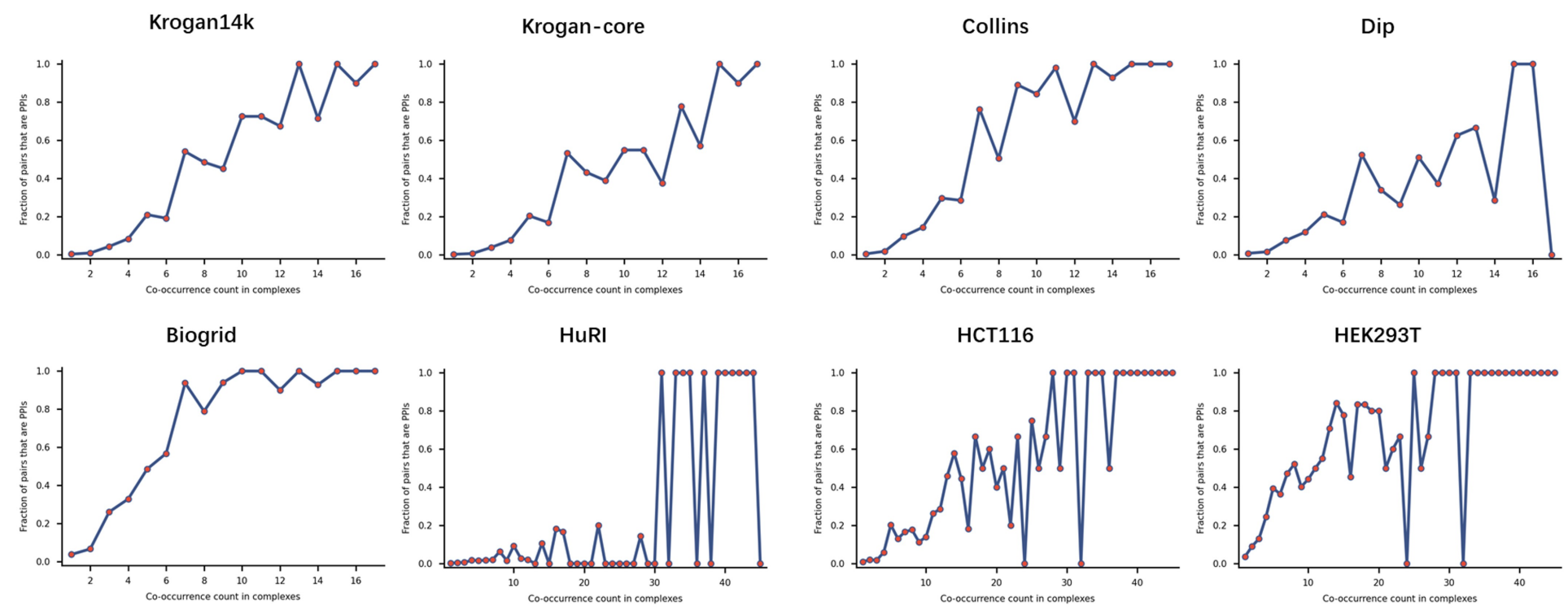

Fig A. Empirical support for Assumption 6.1. For each dataset, we enumerated all protein pairs that co-occur within curated protein complexes and grouped them by their exact co-occurrence count X, where X denotes the number of distinct complexes in which the pair appears together. For each X, we report the fraction of co-occurring pairs that are observed as protein–protein interactions in the corresponding PPI network. Across both yeast datasets, including Krogan14k, Krogan-core, Collins, DIP and BioGRID, and human datasets, including HuRI, HCT116 and HEK293T, pairs that recur across more complexes show higher interaction support. This trend is consistent with the assumption that repeated complex co-membership enriches for biologically grounded physical or functional associations. Fluctuations at large X reflect sparse counts in high co-occurrence bins.

network is incomplete. Deviations and sharp fluctuations at large X are expected, as high co-occurrence bins contain far fewer pairs and are therefore more sensitive to sampling noise and dataset-specific coverage.

7 Code documentation

The source code and data are publicly available at `https://github.com/hyx-1/PCIPG`. To improve reproducibility, we have updated the README file with detailed documentation of the PCIPG implementation. The updated README describes the overall workflow, directory structure, SAGE–GAT model architecture, loss functions, training and inference procedures, evaluation scripts, input/output data formats, tensor shapes, supported datasets, and example command-line usage. These additions provide step-by-step guidance for running the code and reproducing the main experiments.
